# Cytokine modulation in pelvic organ prolapse and urinary incontinence: from molecular insights to therapeutic targets

**DOI:** 10.1186/s10020-024-00989-3

**Published:** 2024-11-13

**Authors:** Yongxiu Chen, Amin Ullah, Weifang Chen, Jianyan Xuan, Xiaowen Huang, Shiqi Liang, Bairong Shen, Tingfeng Wu

**Affiliations:** 1 Department of Neurosurgery, The First Affiliated Hospital of Jinan University, Guangzhou, China; 2 Department of Abdominal Oncology, Cancer Center of West China Hospital and Institutes for Systems Genetics, Frontiers Science Center for Disease-related Molecular Network, West China Hospital, Sichuan University, Chengdu, China; 3 Gynecology Department, Guangdong Women and Children Hospital, Guangzhou, China

**Keywords:** Pelvic organ prolapse (POP), Urinary incontinence (UI), Pro- and anti-inflammatory cytokines, Molecular mechanisms, Therapeutic strategies

## Abstract

Pelvic organ prolapse (POP) and urinary incontinence (UI) are common disorders that significantly impact women’s quality of life. Studies have demonstrated that cytokines, including pro- and anti-inflammatory immune mediators, play a role in illness genesis and progression. Research on the inflammatory milieu of the pelvic floor has shown that POP patients have increased inflammation in vaginal tissues. This evidence revealed that significant changes in the inflammatory milieu of the pelvic floor are an aspect of the pathogenesis of POP. POP patients exhibit increased levels of inflammatory cytokines (IL-1, TNF, IFN, and others) in the front vaginal wall, which may alter collagen metabolism and contribute to POP. Studies indicate that cytokines such as IL-6, IL-10, and TGF, which are involved in inflammation, remodelling, and repair, have dual effects on POP and UI. They can promote tissue healing and regeneration but also exacerbate inflammation and fibrosis, contributing to the progression of these conditions. Understanding the dual roles of these cytokines could help us improve the vaginal microenvironment of women and treat POP and UI. Given the considerable changes in these cytokines, this review addresses studies published between 2000 and 2024 on the molecular mechanisms by which pro- and anti-inflammatory cytokines affect women with POP and UI. Furthermore, we explain novel therapeutic strategies for cytokine regulation, emphasizing the possibility of personalized treatments that address the underlying inflammatory milieu of the vagina in POP and UI patients. This thorough analysis aims to establish a foundation for future research and clinical applications, ultimately improving patient outcomes via designed cytokine-based therapies.

## Introduction

Pelvic organ prolapse (POP) is a significant health concern that affects numerous adult women, and a large population-based cross-sectional study in China revealed a symptomatic prevalence of 9.6% (Pang et al. 2021). Damaged pelvic floor muscles and connective tissue cause the female pelvic organs to drop, leading to a vaginal bulge that can be seen or felt (Donnelly et al. 2024). Stage IV POP seriously impacts women’s social, psychological, and sexual well-being (Kindie et al. 2023). According to an epidemiological investigation, the average incidence of POP is nearly 40%, and the number of people with POP diagnoses has increased over time as the population ages (Hendrix et al. 2002). Currently, the treatment of POP is a severe issue, and surgery serves as the mainstay (Kahn et al. 2022). Hence, a literature review revealed that the lifetime risk of POP surgery is between 12% and 19%. Unfortunately, among women who underwent surgery for all types of POP, 11% underwent reoperation 5 years later, and an additional 4% underwent reoperation 10 years later (Nüssler et al. 2022). Therefore, identifying the molecular profile of POP to establish useful therapeutic options is crucial.

Urinary incontinence (UI), characterized by the involuntary loss of urine, is a prevalent health disease that can cause problems with an individual’s quality of life. This problem affects up to 77% of women (ACOG Practice Bulletin No. 155: Urinary Incontinence in Women 2015; Lukacz et al. 2017; Hu and Pierre 2019; Sussman et al. 2020; Trowbridge and Hoover 2022). Urge urinary incontinence (UUI), mixed urinary incontinence (MUI), and stress urinary incontinence (SUI) are the most prevalent types of incontinence, among several others (ACOG Practice Bulletin No. 155: Urinary Incontinence in Women 2015; Sussman et al. 2020). In the Iranian female population, the prevalence statistics were 20.6% for SUI, 10.4% for UUI, and 8.5% for MUI (Alizadeh et al. 2023). Considering the high incidence of UI in women, early detection and treatment are crucial for mitigating its negative consequences on the quality of life of women through education, prevention, and reducing the impacts of risk factors.

Cytokines are small signalling molecules weighing 10 to − 15 kilodaltons (Lin and Leonard 2019; Silva et al. 2019) that regulate and coordinate host responses to inflammation, infection, and trauma. They restore homeostasis to prevent chronic inflammation. Anti-inflammatory cytokines reduce inflammation and support healing, whereas proinflammatory cytokines cause or exacerbate systemic inflammation (Braz-Silva et al. 2019; Soh et al. 2019). Their modes of action (repair or destruction) rely on their environment and concentrations (Azuma et al. 2014).

**Fig. 1:**
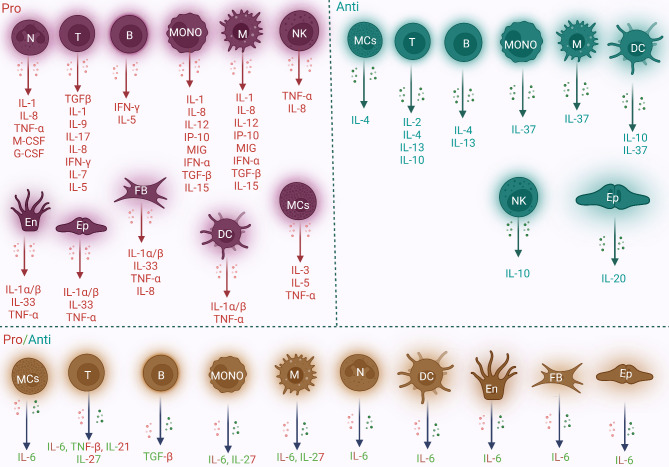
The acronyms “pro-” and “anti-” describe the pro- and anti-inflammatory reactions produced by various immune or nonimmune cells. Proinflammatory cytokines promote inflammation and consequently cause pain and tissue destruction, whereas anti-inflammatory cytokines inhibit the processes that cause inflammation. Inflammatory cytokines support the host’s defence against infection, individually or in combination. This process is known as the chronic inflammatory cytokine response. On the other hand, organ failure and severe tissue damage can result from acute inflammatory reactions. Red cells express proinflammatory cytokines, green cells express anti-inflammatory cytokines, and blue cells express both pro- and anti-inflammatory cytokines, indicating that these molecules may have dual roles. Importantly, the list of cytokines described above is not complete or comprehensive. N, neutrophil; Ep, epithelial cell; B, B cell; T, T cell; M, macrophage; MONO, monocyte; En, endothelial cell; NK, natural killer; FB, fibroblast; DC, dendritic cell; and MC, mast cell

Both immune and nonimmune cells produce these cytokines, which can cause a number of outcomes, including mortality, differentiation, and growth. Tumour necrosis factor (TNF), chemokines, lymphokines, interleukins (ILs), and interferons (IFNs) constitute this group (Silva et al. 2019). In addition, T cells, macrophages, and monocytes secrete significant amounts of proinflammatory cytokines (interleukins). In contrast, T cells produce anti-inflammatory cytokines that reduce inflammation. Figure 1 illustrates the various cell types responsible for the production and secretion of numerous types of cytokines, including those that are proinflammatory, anti-inflammatory, or exhibit both pro- and anti-inflammatory properties (Kany et al. 2019; Amere Subbarao 2021; Bhasin et al. 2022; Wautier and Wautier 2023; Ullah et al. 2024a, b, c). In response to microinjury, inflammatory molecules known as immune mediators or cytokines (interleukins) are critical for inducing inflammatory responses, growth, division, and mortality (Yu et al. 2023). These cytokines alter the mechanical characteristics of pelvic floor tissues by interfering with collagen synthesis in uterus-associated ligament fibroblasts via histiocytes and immune cells (Zhao et al. 2016). The anti-inflammatory molecules IL-10 and IL-4 and the inflammatory mediators IL-1, IL-6, IL-13, prostaglandin E2 (PGE2), and TNF-α are associated with the Janus kinase/signal transducer and activator of transcription (JAK/STAT) signalling pathway through the IL-6 receptor. The stimulation of IL-6 results in a lower collagen content, which further promotes matrix metalloproteinase (MMP) production (Huang et al. 2007; Zhao et al. 2016). Moreover, inflammatory cytokines such as IL-1, IL-6, and TNF-α have been implicated in the pathogenesis of POP through the promotion of extracellular matrix (ECM) component breakdown. In patients with POP, the levels of the proinflammatory cytokines IL-6 and TNF-α positively correlate with MMP1 and MMP2 levels but inversely correlate with TIMP1 levels (Cao et al. 2019; Zhou et al. 2024). Furthermore, a recent study reported that the inflammatory cytokines IL-1β, IL-6, and TNF-α play roles in the pathophysiology of POP (Li et al. 2024b). An evaluation of the inflammatory environment revealed that patients with POP and UI had increased levels of inflammation due to the altered expression of cytokines (Koudounas et al. 2021; Wu et al. 2023b).

**Fig. 2:**
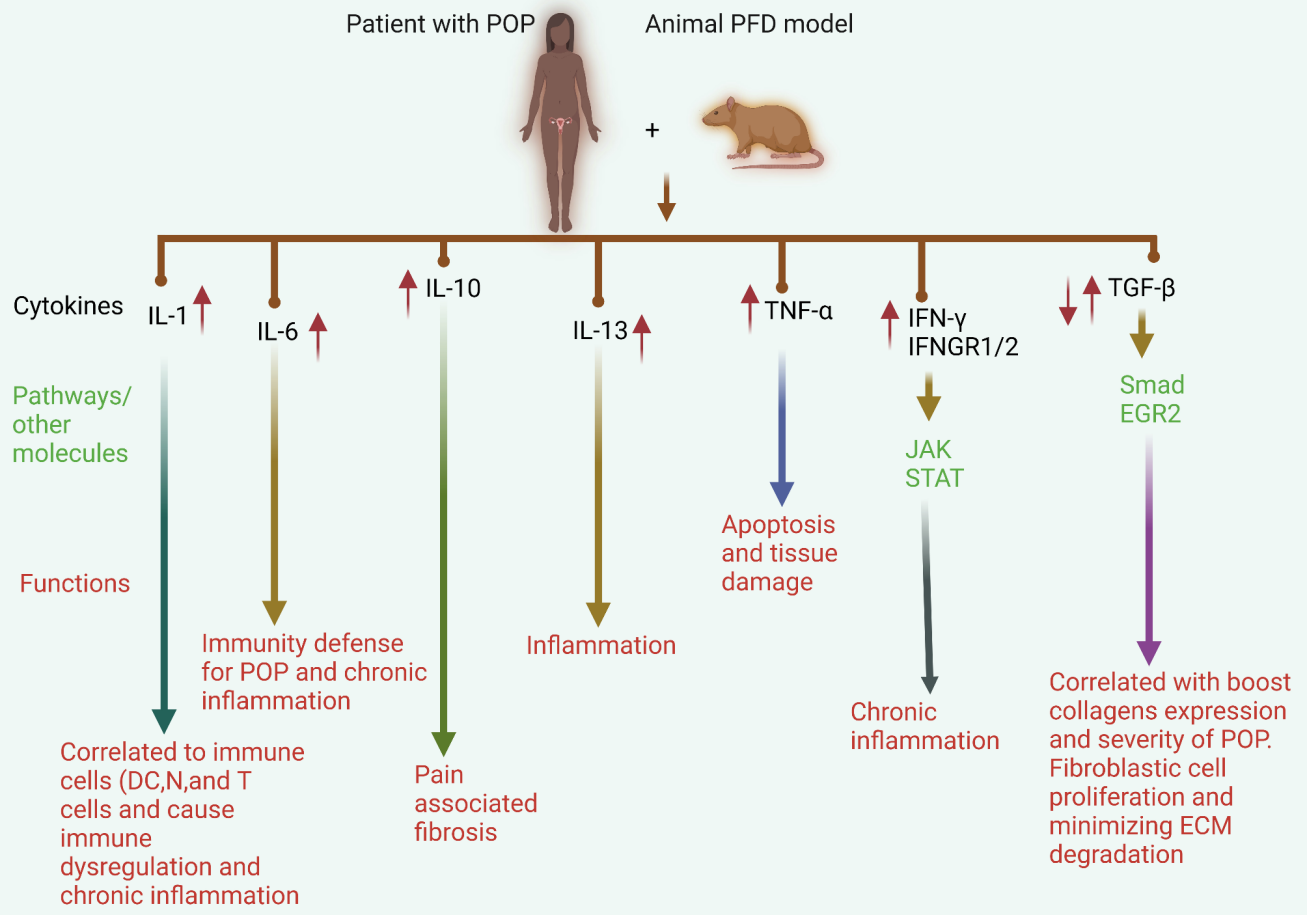
Pro- and anti-inflammatory cytokines are critical in POP development and progression in patients and in PFD animal models. IL-1 is upregulated and associated with many immunological cells, including Ns, DCs, and T cells, resulting in immune dysregulation and chronic inflammation in individuals with POP. A large amount of IL-6 defends against POP and induces chronic inflammation. Moreover, the upregulation of IL-10 induces pain and fibrosis during POP. TNF-α and TRAIL induce ECM remodelling and inflammation via the NF-kB and CASP3 pathways. Dysregulation of TGF-β plays dual roles via the Smad pathway and interactions with EGR2 and collagen molecules. These functions minimize ECM disruption and the severity of POP, fibrosis, and inflammation

Therefore, these studies demonstrate that the expression of cytokines leads to the progression of POP and UI. Thus, speculating about the interactions of these cytokines with POP and UI is logical. Given these findings, the current review focuses on pro- and anti-inflammatory cytokines in POP and UI and the molecular mechanisms that extend beyond these cytokines in clinical and preclinical contexts. An additional section discusses preclinical and clinical studies and describes how these cytokines are potential therapeutic targets in POP and UI. To the best of our knowledge, this comprehensive review is the first to describe the molecular mechanisms underlying pro- and anti-inflammatory cytokines in POP and UI.

## Pro- and anti-inflammatory cytokines and POP

### IL-1

A recent single-cell examination of the uterosacral ligament (USL) revealed cellular heterogeneity in female POP patients. However, the authors also reported that the interaction of the IL-1 family plays a critical role in the genesis and progression of POP by promoting USL collagen deposition in patients with POP (Liu et al. 2024). Wu and colleagues recently elucidated the related mechanisms of pelvic floor-supporting tissue damage caused by mechanical force and the application of stem cell therapy (Wu et al. 2024b). Briefly, this investigation revealed that the accumulation of reactive oxygen species (ROS) in the pelvic floor, which is initiated by mechanical injury, activates the NLR family, pyrin domain-containing 3 (NLRP3) inflammasome, and macrophage pyroptosis, thereby disrupting the immune microenvironment and ECM metabolism. This process leads to chronic inflammation, impaired fibroblast function, and inadequate collagen repair.

**Fig. 3:**
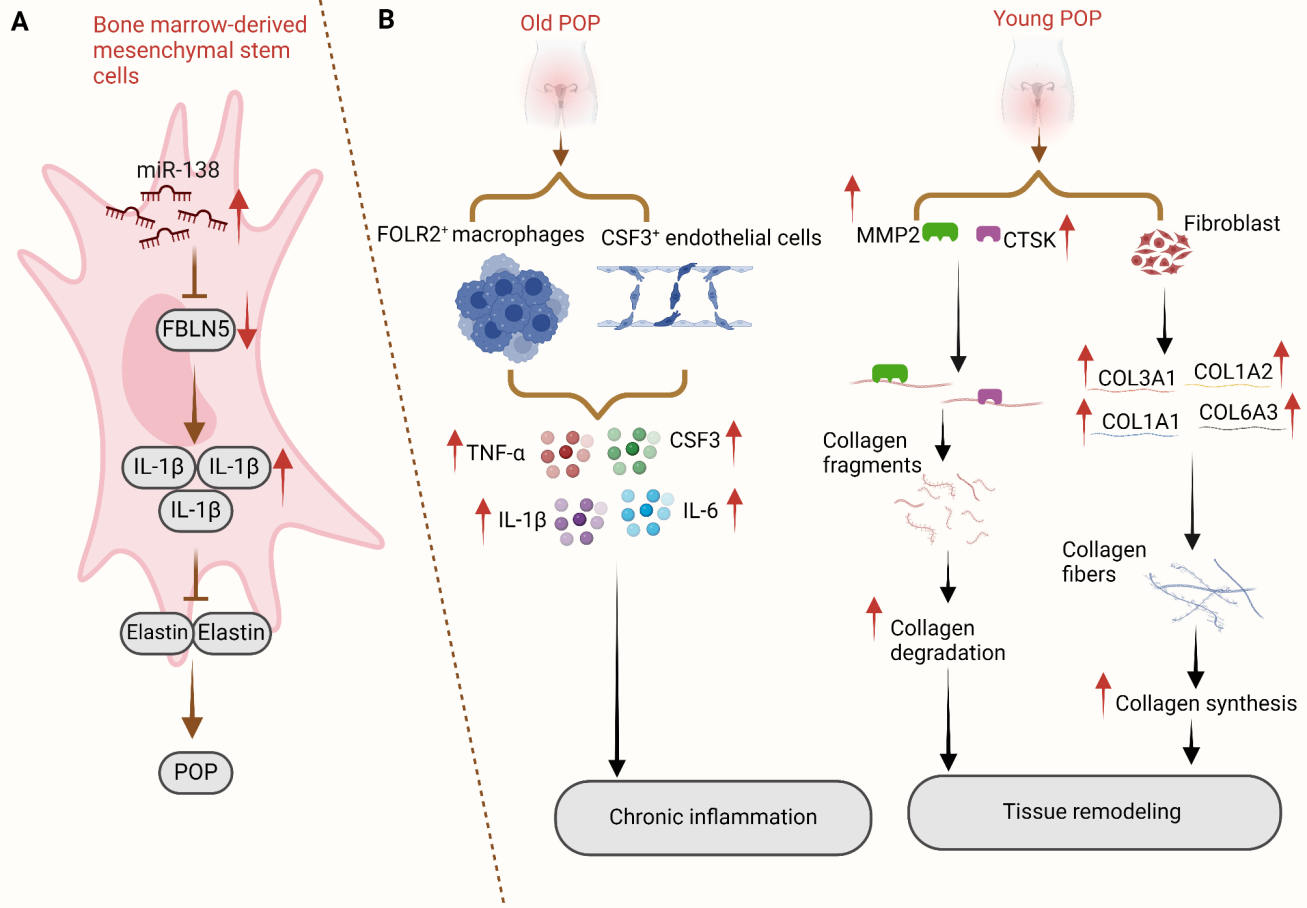
Illustrations of the molecular mechanism underlying the roles of proinflammatory cytokines. (**A**) Through the specific targeting of FBLN5, miR-138 increases the expression of IL-1β, which in turn promotes the progression of POP by inhibiting the expression of elastin. POP in PFD rats decreases after the transplantation of BMSCs with miR-138 silencing. (**B**) The illustration shows the age-related differences in the pathophysiology of young and old patients with POP. In old patients with POP, chronic inflammation is the primary cause of the pathology, whereas in young patients with POP, tissue remodelling is the primary cause. Following chronic inflammation, aged patients with POP exhibit the release of inflammatory cytokines, including TNF-α, IL-6, and IL-1. Conversely, samples from young patients with POP activate extracellular matrix metabolism and increase the expression of MMP2, CTSK, and other genes. These findings suggest that POP may be associated with the dysregulation of numerous genes, as distinct age groups present different gene expression trends

Following acute mechanical injury, ROS accumulate in pelvic floor tissues, attracting macrophages. ROS stimulate M1 macrophages to release the NLRP3 inflammasome and IL-1β, inducing pyroptosis and exacerbating tissue inflammation. This chronic inflammation impairs fibroblast adhesion, movement, and activity, which causes an imbalance in the collagen fibres of the ECM and impedes the healing process. Furthermore, stem cells may provide therapeutic benefits by reducing ROS accumulation and pyroptosis through their antioxidant capacity and promoting M1 to M2 macrophage polarization. This process reduces proinflammatory factor release and facilitates tissue reconstruction (Fig. 3A) (Wu et al. 2024b). Additionally, stem cells release growth factors such as fibroblast growth factor, stimulating collagen synthesis and tissue repair. The authors developed an ROS-responsive stem cell hydrogel (PVA@COLI) to improve stem cell survival and function and to enhance the therapeutic effect. This hydrogel acts as a protective barrier, maintaining cellular activity and reshaping the inflammatory microenvironment by clearing ROS, thus promoting the repair and regeneration of the injured pelvic floor tissue (Fig. 3A) (Wu et al. 2024b). This approach holds promise for effective tissue repair in patients with POP.

Prior research on the host response to mesh has used identical mesh sizes for all the study groups. Prudente et al. conducted studies involving the implantation of mesh into the abdominal subcutaneous tissue of rats and reported elevated inflammatory responses (higher but not significantly higher IL-1 levels) in the early postimplantation phase (Prudente et al. 2016). Similarly, Bronzatto and Riccetto compared the implantation of standard and lightweight meshes and observed elevated expression of MMP-2, MMP-3, and IL-1 on Day 30, which may indicate a chronic inflammatory response (Bronzatto and Riccetto 2018). The mesh density did not affect the levels of the proinflammatory cytokines IL-1 and TNF-α or the levels of MMPs 2 and 3. The increased expression of IL-1, MMP-2, and MMP-3 over time may indicate a long-term inflammatory response to polypropylene (PP) mesh implantation. Factors other than the amount of the implanted material may influence adverse effects after PP prosthetic implantation.

In diabetic patients, IL-1α is paradoxically downregulated following mesh removal and is also involved in a mesh biorepository (Liang et al. 2024). Furthermore, both groups showed strong positive correlations between IL-1 expression and several cell type-specific markers, including markers of regulatory T cells (Foxp3), neutrophils (Fcgr3b), and dendritic cells (DCs) (Cd80, Cd1a, Cd1b, Cd1e, Flt3, and Fosl1). Notably, a significant positive association was observed between Fosl1 expression and IL-1a levels. Only the diabetic group, not the nondiabetic group, presented a positive correlation with the expression of DC markers (Cd80, Cd1b, Cd1e, and Flt3), suggesting that DCs play a role in downregulating IL-1a in the glycaemic environment (Liang et al. 2024). The surprisingly downregulated expression of IL-1a in the diabetic group indicates impaired immunity against infection. Diabetic women are more likely to experience mesh difficulties due to the potential for both types of aberrations to prevent proper integration of the mesh into the tissue (Liang et al. 2024). Diabetes affects the expression of pro-healing DC indicators such as IL-1a, suggesting that these crucial innate immune cells may contribute to diabetes-associated immunological dysregulation. Targeting these innate immune cell types may improve mesh outcomes in diabetic women, but more in-depth investigations are needed.

Researchers have conducted urodynamic and immunohistochemical analyses 60 and 180 days after mesh removal. The findings indicated a significant increase in the levels of inflammatory mediators (TNF and IL-1) in the entire mesh removal group after 60 days (M-T 60D) (Lo et al. 2022). The extent of the procedure is correlated with an inflammatory reaction. These findings imply that complete mesh excision causes a host inflammatory response and transient lower urinary tract problems.

**Fig. 4:**
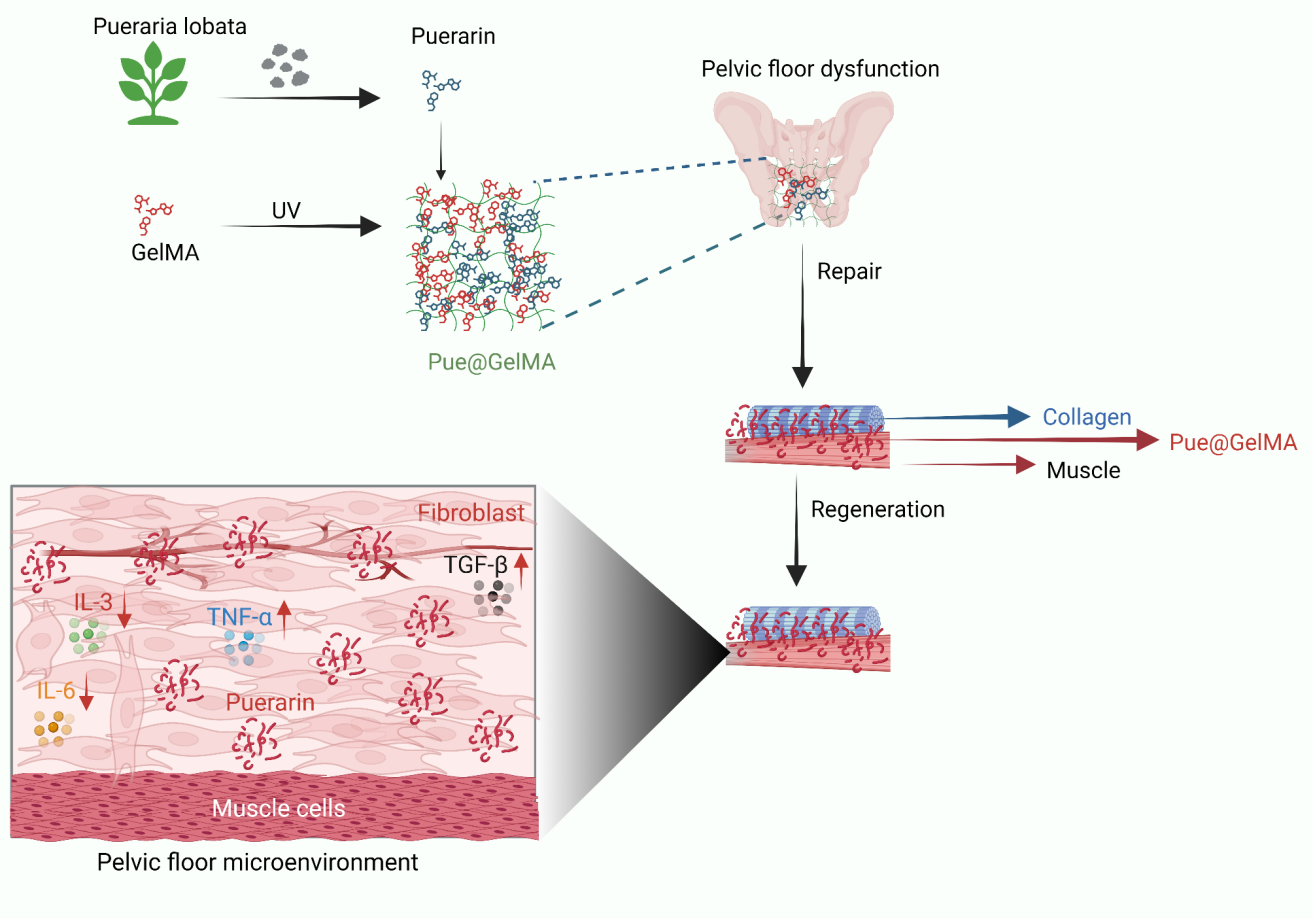
Illustration of the molecular mechanism of GelMA-mediated pelvic floor fascia repair and the surgical process of POP repair. Pue@GelMA, an anti-inflammatory biomaterial, reduces the levels of inflammatory cytokines, enhances fascia regeneration via the TGF-β/MMP pathway, and accelerates pelvic floor tissue restoration. Pue@GelMA reduces early-stage inflammation by lowering TNF-α, IL-3, and IL-6 expression and promoting fibroblast adhesion and development on hydrogels. In addition, the prolonged release of Pue increases the production of type I collagen and helps the fascial tissue of the pelvic floor heal and grow faster in gynaecology. Pue stimulates long-term fibroblast proliferation and collagen deposition on the ECM to achieve high-strength restoration of the pelvic floor fascia

Researchers discovered that miRNAs are linked to the hub genes in the miRNA–hub gene network and regulate the hub gene expression during POP (Zhou et al. 2023). Previous basic research has revealed that miRNA-138 can influence the expression of crucial genes (Fig. 4A) (Zhao et al. 2021a). Briefly, the authors developed a rat pelvic floor dysfunction (PFD) model and used leak point pressure (LPP) and conscious cystometry (CMG) to assess the effectiveness of a BMSC injection. MiR-138 inhibition improved the survival of bone marrow mesenchymal stem/stromal cells (BMSCs) and increased elastin secretion while reducing the expression of IL-1β (Zhao et al. 2021a). In vivo, BMSCs treated with miR-138 inhibitors presented improved LPP and conscious CMG outcomes. The use of miR-138 could be an intriguing therapy for addressing POP via tissue engineering.

### IL-6

A recent study revealed that the majority of individuals with POP have increased levels of proinflammatory cytokines (IL-6) (Ekwedigwe et al. 2020). Recent bioinformatic research has revealed robust infiltration of immune cells, including macrophages, monocytes, Tregs, Th1 cells, and NKT cells, in POP tissues. Furthermore, POP is closely associated with LOX, IL-6, SDC1, ICAM1, and CD38 levels. The authors stated that the ECM and immune-related signature genes, together with extensively infiltrating immunocytes, may play a role in the development of POP (Wu et al. 2023a). Similarly, microarray data and bioinformatics analysis have been used to identify significant genes, including IL-6, associated with the onset and progression of POP (Zhou et al. 2018). In addition, research has demonstrated that specific inflammatory genes, including IL-1β, IL-6, and TNF-α, are prominently expressed in endothelial cells, fibroblasts, and macrophages in older populations with POP. These findings suggest a significant enrichment of inflammation-related genes in this demographic group. Conversely, younger patients with POP present distinct expression profiles, with endothelial cells, fibroblasts, and macrophages highly expressing COL3A1, MMP11, and S100A8, indicating an enrichment of genes associated with ECM metabolism (Li et al. 2021; Miao et al. 2023). These findings suggest that older patients with POP experience biological processes that contribute to chronic inflammation, whereas younger patients exhibit processes related to ECM metabolism and potential immune regulation (Fig. 4B) (Miao et al. 2023). Furthermore, specific gene expression patterns have been identified in certain cell subtypes. For example, subtype 1 endothelial cells highly express the inflammatory factor CSF3, leading to their classification as CSF3 + endothelial cells. Similarly, subtype 2 macrophages, classified as FOLR2 + macrophages, present elevated levels of FOLR2 (Miao et al. 2023). These results also suggest that the presence of CSF3 + endothelial cells and FOLR2 + macrophages may be closely associated with POP in older individuals and could represent a cytological basis for the observed increase in inflammatory processes in this population. Therefore, the biological processes or molecular mechanisms associated with inflammation in POP need to be further investigated in aged populations.

Furthermore, a recent study revealed that the levels of proinflammatory markers, including TNF-α and IL-6, were higher during forceps-assisted birth than during spontaneous vaginal delivery. On the other hand, compared with women who underwent caesarean delivery, the levels of the anti-inflammatory factors IL-4 and IL-10 are higher in women who underwent natural delivery via the vagina (Han et al. 2023). These findings suggest that caesarean section exacerbates perinatal stress and inflammatory reactions in high-risk pregnant women and link the mode of delivery to postpartum stress and inflammatory responses. Thus, safe delivery methods, appropriate forceps, and preventive measures should be used in high-risk pregnant women to reduce postpartum infection, protect the pelvic floor, promote vaginal instrument midwifery, and prevent excessive pelvic floor extension. Previously, Brizzolara and colleagues (Brizzolara et al. 2009) reported an increase in the activity of several immune- and defence-related genes. These genes included the IL-6, Toll-like receptor, and interferon (IFN)-γ receptor (IFNGR) genes. These findings suggest that these genes, which are enriched for “immunity and defence,” cause POP.

### IL-10 and IL-13

IL-10 is considered an anti-inflammatory cytokine with pleiotropic functions, and its effects depend on its concentration. Under physiological conditions, it has anti-inflammatory properties. However, IL-10 can induce fibrosis when it is overexpressed, as shown by elevated TGF-β levels in the pulmonary tissues of IL-10 transgenic mice (Lee et al. 2002). In vivo data from animal studies and analyses of mesh explanted from the pelvic floor of symptomatic patients also highlight the role of IL-10 in fibrosis. In a study by Brown et al., three different polypropylene meshes of different weights and porosities were implanted into rhesus macaques (Brown et al. 2015). These meshes were explanted 12 weeks later, and the macrophage response was studied. The histologic evaluation revealed a profibrotic response in the lighter, more porous meshes with significantly increased IL-10 levels compared with a proinflammatory response in the heavier, less porous mesh. Similarly, a recent study reported that macrophages exposed to a mechanochemically distressed PP mesh significantly upregulate the expression of IL-10, a profibrotic marker (Farr et al. 2024). This observation suggests that macrophages are activated by mechanical stress, which results in the development of a fibrotic environment. Although IL-10 is typically associated with anti-inflammatory properties, its coexistence with pro-fibrotic markers such as TGF-β and IL-13 implies a complex interaction in which inflammation and fibrosis may manifest simultaneously (Farr et al. 2024), highlighting the potential role of IL-10 in the fibrotic response. In addition, a recent study examined the gene expression profiles of primary vaginal fibroblasts from two groups: those with POP and those without POP (Xu et al. 2024). Using gene set enrichment analysis (GSEA), researchers identified key biological pathways linked to disease progression. These pathways involve cell proliferation, ECM remodelling, and inflammatory processes, with a specific focus on the ECM degradation and signalling pathways activated by IL-4 and IL-13. Furthermore, Nolfi and colleagues (Nolfi et al. 2016) examined PP vaginal explants removed after 4.5–93 months. They observed links between patient-reported pain and profibrotic interleukin-10 levels and M2 “remodelling” macrophages, which cause the pain associated with fibrosis. The proteolytic activity of MMP-9 increased in patients with further erosion, indicating degradation. Consequently, these mechanisms correlate with pain and mesh exposure, which may impact different levels of macrophage activation. Thus, future studies will need to focus on specific risk factors associated with certain types of mesh challenges.

### TNF-α and IFN

A recent study assessed tissue remodelling and inflammatory responses. After mesh implantation and paravesical space dissection, the MF study group experienced considerable increases in the levels of inflammatory mediators such as IL-1 and TNF-α (Lo et al. 2020). The inflammatory response predicts the recruitment and activation of more inflammatory cells, which leads to cell apoptosis and tissue damage (Lo et al. 2017), contributing to the emergence of symptoms related to the lower urinary tract, such as a reduced voiding interval and low LPP, in this specific group of rats (Study MF); these results support clinical practice notions of an association between lower urinary tract symptoms and opening the paravesical space.

In the vaginal tissue of premenopausal women with POP, Zhao and colleagues (Zhao et al. 2014) reported increased expression of IFN-γ and its receptors, IFNGR1 and IFNGR2. Researchers have also reported elevated mRNA expression of IFNγ, IFNGR, and IFNGR2 in the uterine tissues of POP patients. Furthermore, they reported upregulated mRNA expression of genes involved in the JAK-STAT signalling pathway, which contributed to the activation of IFNγ (Zhao et al. 2014). These findings suggest that inflammatory cytokines, such as IFN-γ and POP, are closely associated with each other. Therefore, whether cytokines regulate the development of POP or result from the tissue prolapse-induced chronic inflammatory response remains unclear. Thus, more research on the molecular mechanism is needed.

### Dual roles of TGF

The profibrotic cytokine TGF-1 significantly influences degenerative fibrotic disease. This cytokine can transform cells from various organs into fibroblasts, in addition to promoting the release of paracrine and autocrine growth factors and formation of the ECM. TGF-β1 is essential for the metabolism of the ECM and the proliferation of fibroblasts (Sampson et al. 2014). At the cellular level, TGF-β1 binds to its receptor TRβII (TGF-β receptor II), a process that is facilitated by the suppressor of the mother against decapentaplegic (Smad) transcription factors. Due to their roles in signal transduction from TRβ2 to the nucleus, these transcription factors from the Smad family are crucial for the TGF-β1 signalling pathway (Moustakas and Heldin 2005; Niu et al. 2008). Upon activation, TGF-β1 plays a critical role in the ECM remodelling process and fibrogenesis, regulating the expression of MMPs and TIMPs and the deposition of critical ECM components. The process of collagen degradation is inhibited by TGF-β1, which increases TIMP expression and blocks the actions of MMPs. Additionally, it stimulates fibroblast differentiation and the synthesis of collagens, particularly type I and II collagens, by myofibroblasts (Gressner and Weiskirchen 2006; Liu et al. 2017). However, previous studies have revealed the dual roles of TGF-1 in POP.

TGF-β1, a typical multifunctional cytokine that generally regulates numerous biological functions and has been implicated in the synthesis of connective tissue, is the focus of research in the field of collagen synthesis (Yang et al. 2010). The degree of POP is correlated with decreasing TGFβ1 mRNA and protein expression levels (Liu et al. 2017). Pretreatment with TGF-β1 attenuates the loss of ECM by activating the TGF-β1/Smad signalling pathway (Min et al. 2017a). TGF-β1 promotes collagen synthesis through the following mechanisms, as determined by an examination of its mechanistic contribution to chronic kidney diseases and idiopathic pulmonary fibrosis: (i) activation of downstream signalling pathways, including the TGF-β1/Smad pathway; (ii) increased de novo synthesis of serine; and (iii) increased expression of protease inhibitors (Meng et al. 2016; Nigdelioglu et al. 2016). Speculation that downregulating TGF-β1 impedes collagen synthesis, disrupts ECM metabolism, and ultimately influences the occurrence and progression of POP is feasible. These results indicate that TGF-β1 has a synergistic effect on collagen expression. A recent study revealed that these two signalling mediators play significant roles in the development of the ECM (Zhang et al. 2020b). Maintaining the mechanical strength of supportive pelvic connective tissues through the balanced turnover of collagen is crucial.

The primary MMPs are MMP1, 2, 3, and 9. These enzymes can degrade collagen and other components of the ECM (Cui et al. 2017). A prior investigation revealed that the overexpression of MMP2 is a risk factor for POP (Phillips et al. 2006). MMP2/9 expression levels in pelvic floor tissue are increased in POP patients, whereas the expression levels of TIMPs—which inhibit MMP activity—are decreased (Ma et al. 2012). Thus, MMPs and TIMPs are thought to be the primary regulators of ECM degradation. Recent studies have shown that POP patients have significantly higher levels of TGF-1 and MMP-3 mRNA expression than non-POP patients do, indicating the severity of POP (Chen et al. 2024b). Meijerink and colleagues reported a positive correlation between POP and TGF-β1 levels in the vaginal wall(Meijerink et al. 2013). Sun and colleagues (Sun et al. 2014) suggested that TGF-β1 may have a functional impact on POP patients by affecting fibroblast proliferation. Moreover, the TGFβ1/Smad3 signalling pathway reduces the loss of ECM by inducing the production of TIMP-2 and inhibiting the activity of MMP2/9 (Liu et al. 2017; Min et al. 2017a). Nevertheless, in another study that used a rainbow trout cardiac fibrosis model, TIMP-2 and MMP9 transcript levels increased 24 h after receiving TGF-β1 therapy (Johnston and Gillis 2017).

According to a 2013 study, the disruption of the equilibrium between oxidation and antioxidants in females with POP resulted in an increase in the levels of ROS and a downregulation of the TGF-β1 signalling pathway. These changes led to damage to the pelvic support structure and the inhibition of collagen synthesis (Li et al. 2013). Furthermore, reduced oxidative stress (OS) may facilitate collagen synthesis. In contrast, increased OS may disrupt the homeostasis and metabolism of the ECM by influencing the levels of MMPs/TIMPs and the activation of the TGF-β1/Smad signalling pathway. This process, in turn, leads to the induction of POP (Stewart et al. 2018). Liu and colleagues (Liu et al. 2016) studied OS in patients with collagen-associated illnesses that contribute to POP. They examined parametrial ligament samples from 40 hysterectomy patients separated into two groups: POP patients (stage II–IV) and controls. The samples were treated with H2O2 (0.1 to 1 mM) for 4 to 24 h, and OS damage was assessed by immunohistochemistry for 8-OHdG and 4-HNE, which were present at higher levels in POP patients. The results revealed that H2O2 toxicity was concentration and time dependent, with considerable cytotoxicity and apoptosis. The expression of COL1A1, MMP2, TIMP1, and TGF-β1 was measured to assess collagen metabolism. Higher H2O2 concentrations reduced COL1A1, TIMP1, and TGF-β1 expression while increasing MMP2 expression. Furthermore, the authors discovered that lower H2O2 concentrations increased COL1A1 production (anabolic effect), whereas higher H2O2 concentrations increased catabolism. OS was found to impair collagen homeostasis, which contributes to POP (Liu et al. 2016). Increasing H2O2 concentrations decreased TGF-β1 expression, affecting collagen modulation and aggravating POP. The study hypothesized that OS affects the MMP/TIMP balance and interferes with the TGF-β1/Smad pathway, reducing elastic fibre formation and diminishing pelvic floor support, ultimately resulting in POP. In addition, given that the ECM plays a significant role in POP pathogenesis, a 2017 (Zhang et al. 2017) study examined the effects of high levels of mechanical stress on human uterosacral ligament fibroblasts (hUSLFs), as well as their exposure to H2O2. Tissue samples were collected from 15 individuals who underwent vaginal hysterectomy for nonneoplastic diseases and were subjected to different levels of mechanical stress and exposure to various concentrations of H2O2. These treatments contributed to considerable decreases in mitochondrial DNA and ECM protein expression. Lower H2O2 concentrations increased the expression of ECM proteins while inhibiting the expression of elastin and many collagen precursors (COL 1A1 and COL 3A1). This result was also observed when weaker forces were applied to the hUSLF tissue samples. Another interesting observation is that MMP levels increased while TIMP expression decreased, facilitating ECM destruction. The authors studied the effects of exposure to excess H2O2 and mechanical stress on the Smad/TGF-β1 pathway. The expression of phosphorylated Smad2 and activated TGF-β1 decreased. The authors suggested that high levels of mechanical stress and exposure to excess H2O2 interfere with the Smad/TGF-β1 pathway, significantly altering ECM remodelling and promoting POP (Zhang et al. 2017). Kluivers and colleagues (Kluivers et al. 2023) conducted a recent study that established a molecular landscape of POP. This landscape was based on a large exome chip study. Their research identified four primary biological processes in POP: the epithelial‒mesenchymal transition, immune response activation, ECM modulation, and fibroblast survival and apoptosis. They demonstrated that the ECM supports both epithelial cells and fibroblasts, with TGF-β contributing to the remodelling of the ECM, indicating its potential as a therapeutic target.

Furthermore, POP fibroblasts with early growth response protein 2 (EGR2) knockdown had improved cell proliferation and PCNA expression and reduced apoptosis. Researchers revealed that EGR2 knockdown led to increased COL1A1, COL3A1, and elastin expression; decreased MMP2 and MMP9 activities; and increased TGF-β/Smad pathway activity in POP fibroblasts (Jin et al. 2024). Interestingly, dual-luciferase experiments revealed that EGR2 increased SOCS3 transcription. EGR2 knockdown reduced SOCS3 expression, improved POP fibroblast proliferation and collagen synthesis, and decreased apoptosis (Jin et al. 2024). Overall, this study showed that POP tissues presented high expression levels of EGR2 and that EGR2 knockdown mitigated POP fibroblast apoptosis and matrix degradation. This research may provide a new perspective on the pathophysiology of POP.

In contrast to the above studies, neither TGF-II expression nor TGF-I1 expression changed between USL samples and non-POP controls (Leegant et al. 2015; Vetuschi et al. 2018; Ying et al. 2022). However, according to numerous studies conducted recently(Qi et al. 2011; Carlin et al. 2020; Zhao et al. 2021b), TGF-β1 expression is negatively correlated with POP or its different stages. In the studies above, the distinct outcomes observed with respect to TGF-β expression and its functions are influenced by variations in research methodologies, material locations, study populations, sample sizes, and genetic backgrounds.

## Pro- and anti-inflammatory cytokines and urinary incontinence (UI)

### IL-1

Pillalamarri and colleagues (Pillalamarri et al. 2017) reported the use of urine cytokines to examine patients with overactive bladder (OAB) accompanied by UI for infection-induced inflammatory biomarkers. Alterations in cytokine and chemokine profiles in OAB patients’ urine suggest an infectious or inflammatory state, as IL-1β expression is linked to worsening symptoms (Pillalamarri et al. 2017). Thus, a large-scale trial with well-characterized patient cohorts is needed to establish the incidence and relative changes in urine cytokine levels in patients with OAB. In addition, inflammatory cytokines (IL-1α, IL-1β, TNF-α, and IL-8) are released when the skin of healthy volunteers is exposed to synthetic urine and stool. Exposure to synthetic urine was linked to an elevated level of IL-1α, whereas exposure to synthetic stool was linked to a higher level of TNF-α (Koudounas et al. 2021). These results showed that TNF-α and IL-1α are potential biomarkers for identifying skin injury when incontinence occurs. Furthermore, previous studies revealed that the type II interleukin-1 receptor (IL-R2) is downregulated in patients with SUI (Chen et al. 2003). This downregulated gene can mitigate the effects of IL-1 on the production of proinflammatory mediators, matrix MMP activity, and proteoglycan synthesis (Amin 2000).

**Fig. 5:**
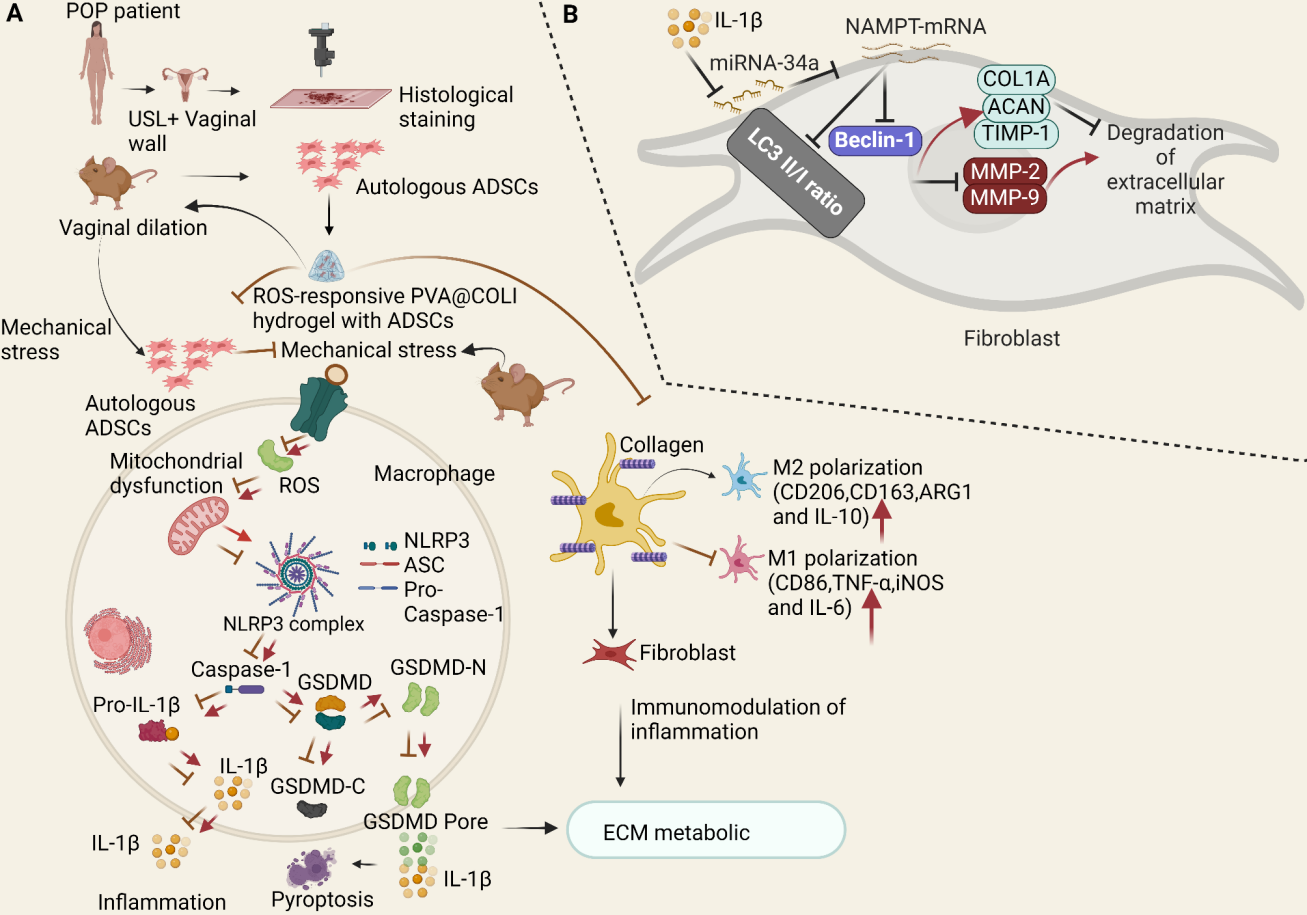
Diagrams of physiological processes underlying the roles of proinflammatory cytokines. (**A**) Schematic showing the therapeutic effects of adipose mesenchymal stem cells (ADSCs) on mechanically induced pelvic floor tissue damage and the production of hydrogels that respond to ROS to treat injury. Many ROS and macrophages quickly accumulate in pelvic floor tissues following mechanical stress, both in vivo and in vitro. ROS cause ECM collagen metabolic disorders in the pelvic floor. These molecules prompt macrophages to form the NLRP3 inflammasome, which results in the release of IL-1β and pyroptosis, resulting in the inflammation of damaged tissues. Additionally, ROS prolong the chronic inflammation of fibroblasts in supporting tissues. A ROS-responsive PVA@COLI hydrogel was simultaneously combined with ADSCs to overcome the low cell survival rate and poor therapeutic effect of a direct cell injection. The ROS-scavenging characteristics of the gel may modify the specific site of inflammatory injury, improve cell survival, and assist in subsequent treatment. (**B**) A schematic of the mechanism through which miR-34a modifies the ECM metabolism of FVWFs in individuals with SUI through Nampt-mediated autophagy. IL-1 treatment decreases the expression of miR-34a in FVWFs, and by blocking Nampt transcription, miR-34a suppresses ECM breakdown and accelerates autophagy

MiR-34a upregulation suppressed SUI in IL-1β-treated FVWFs by increasing collagen and TIMP-1, lowering MMP-2 and MMP-9, and blocking nicotinamide phosphoribosyltransferase (Nampt) transcription, which accelerated autophagy and suppressed ECM breakdown (Zhou et al. 2022). In functional rescue studies, Zhang and colleagues (Zhang et al. 2022a) recently showed that suppressing autophagy is vital for understanding its role in the modulation of IL-1-treated fibroblast ECM breakdown. Patients with SUI, animal models, and fibroblasts treated with IL-1 presented high expression of Nampt. However, Nampt silencing inhibited ECM breakdown and increased SUI fibroblast autophagy. Additionally, autophagy inhibition reduced the inhibitory effects of Nampt silencing on ECM degradation in SUI fibroblasts. As a result, the authors discovered that SUI fibroblasts overexpressed Nampt, and inhibiting it increased SUI fibroblast autophagy and reduced ECM breakdown (Fig. 5B) (Zhang et al. 2022a). However, more research is needed to determine whether Nampt mediates ECM breakdown via alternative molecular pathways. Additionally, further research is necessary to fully understand the effects and other roles of Nampt in SUI and to determine the effective use of Nampt silencing in clinical settings. However, these findings provide new insights into the field of SUI research.

Moreover, patients with UI and hypersensitive bladder (HSB) have decreased levels of IL-2 and increased (not statistically significant) levels of IL-1β (Chen et al. 2023). Patients with both HSB and detrusor overactivity (DO) may also have an inflammatory bladder environment because both conditions have similar symptoms. The variations, as well as associations between DO and HSB with regard to the aetiology and therapeutic outcomes, require further investigation.

### IL-5 and IL-6

According to a recent publication by Firouzmand and colleagues (Firouzmand et al. 2020), the IL-5 levels has served as a biomarker for patients with UI symptoms associated with OAB. As a result, IL-5 testing may promote a more individualized approach to treatment through the development of new noninvasive diagnostic tests for various OAB inflammatory phenotypes. Rechberger and colleagues (Rechberger et al. 2009) studied preoperative cytokine levels and PP mesh erosion after suburethral sling implantation. Patients with and without mesh erosion (SUI) were examined for preoperative serum cytokine levels. The authors reported that mesh erosion was associated with significantly higher pretreatment levels of IFN and IL-5 (not significantly different) than normal vaginal wound healing. This result implies that an excessive systemic response could cause mesh challenges.

Mesenchymal stromal cell (MSC) treatment also has poor efficacy in treating UI, a condition prevalent in women with diabetes and obesity. Researchers discovered that long-term exposure to a dyslipidaemic environment alters the epigenetic composition of genes, including IL-6, myostatin, and microRNAs, leading to aberrant global transcriptional features. Additionally, this disruption affects the ability of MSCs to repair muscle tissue (Kovanecz et al. 2019). In patients with obesity and diabetes, fat infiltration, apoptosis, and the inability to repair the injury depend on the myostatin/IL-6 balance, which may be critical in causing stem cell damage. This result needs to be confirmed.

### IL-10

Serotonin, a hormone, regulates proinflammatory cytokines and affects maternal–foetal hyperglycaemic pathways (França et al. 2023b). Furthermore, IL-10 can modulate serotonin transporter function and serotonergic behaviours (Latorre et al. 2013). A recent study revealed that pregnant women with gestational diabetes and UI released higher levels of IL-10 in their urine but had lower levels in their plasma (França et al. 2023a). Notably, pregnant women with gestational diabetes and UI had higher levels of IL-10 in their urine, and a positive correlation was observed between plasma and urine levels in this group. Furthermore, these urine levels of serotonin and IL-10 were positively correlated in these patients (França et al. 2023a). These results suggest that hyperglycaemia, particularly when accompanied by involuntary urine loss, may inhibit the synthesis of IL-10 in the body. Decreased serotonin levels and increased excretion of cytokines in the urine may be linked to this reduction in IL-10 levels, which might disturb anti-inflammatory processes and increase inflammation. Therefore, more research is needed to confirm the processes underlying alterations in blood and urine levels of serotonin and IL-10 and the modulatory functions of these bioactive elements in gestational diabetes, which is associated with pregnancy-related UI. Similarly, during UI, patients with OAB have lower levels of the anti-inflammatory cytokine IL-10 (Pillalamarri et al. 2017). Women with OAB may have a proliferative inflammatory response due to a shift in the balance of these cytokines towards a more proinflammatory state and the absence of anti-inflammatory cytokines, which could contribute to the pathophysiology of this disease.

### TNF

Momozono and colleagues reported that TNF-α expression in robot-assisted radical prostatectomy (RARP) samples accurately corresponded with urethral fibrotic alterations and may have predicted the continence status early after surgery (Momozono et al. 2016). Thus, urethral fibrosis due to inflammation (TNF-α) may be a risk factor for incontinence among certain demographic groups following radiation treatment. Moreover, in a dose-dependent manner, TNF-α inhibited the myogenic development of human urethral rhabdosphincter cells via the phosphoinositide 3-kinase (PI3K) and p38-mitogen-activated protein kinase (MAPK) pathways, indicating that TNF-α could be a risk factor for SUI in elderly individuals (Shinohara et al. 2017).

Simulated birth trauma causes SUI and urethral dysfunction in rats with vaginal distention (VD) (Yoshikawa et al. 2017). Yoshikawa and colleagues (Yoshikawa et al. 2017). In the single VD model, the urethral baseline pressure (UBP) and the amplitude of the urethral response to electrical stimulation (A-URE) decreased significantly four days after surgery in all the VD model rats. Moreover, TNFR, TNF⍺, and IL-6 levels increased after birth trauma, indicating persistent urethral inflammation; exposure to several types of muscle trauma appeared to have various effects on these markers (Yoshikawa et al. 2017). The results from rats confirmed that the negative effects of VD depend on the evaluated pelvic floor, which could be related to each individual’s plastic adaptation during pregnancy and the postpartum period (Alperin et al. 2015).

### TGF and Smad

Studies on TGF expression in patients with UI have reported conflicting findings. Human studies investigating differences in TGFB1 protein expression revealed decreased (Li et al. 2019a) and no abnormal differences in protein expression (Wen et al. 2006), and a study measuring serum TGFB1 levels in SUI patients compared with controls similarly revealed no abnormal differences (Suzme et al. 2007). Furthermore, animal investigations revealed inconsistent findings regarding Tgfb1 levels (Li et al. 2012; Min et al. 2017b; Tang et al. 2017a, b; Zhang et al. 2020a) in SUI models compared with controls. These discrepancies may be attributed to differences in the study populations, methodologies, or stages of UI being investigated. Future research should aim to standardize protocols, include diverse patient cohorts, and explore the molecular mechanisms underlying the role of TGF-β1 in subjects with different incontinence subtypes to clarify these inconsistencies and guide targeted therapeutic strategies.

TGF-β1 promotes cell proliferation, differentiation, and survival, facilitating injury repair processes (Lin et al. 2010). It regulates the ECM by phosphorylating Smad2 and Smad3 and stimulating the expression of ECM components such as collagen, fibronectin, and elastin (Samarakoon et al. 2013). A recent study examined the impact of ginsenoside Rb1 (GS-Rb1, which has potential antioxidant and anti-inflammatory activities) on the levels of TGF-β1 and associated proteins. Compared with those of control and GS-Rb1-treated rats, the urethral tissues of SUI rats presented lower levels of TGF-1, Smad3, p-Smad3, and collagens I and III (Chen et al. 2024a).

Furthermore, the authors reported that GS-Rb1 stimulated the expression of TGF-β1, Smad2, Smad3, Smad7, phosphorylated Smad3 (p-Smad3), phosphorylated Smad2 (p-Smad2), and collagens I and III. These findings suggest that GS-Rb1 activates several elements of the TGF-β/Smad signalling pathway, which are crucial for the synthesis and regulation of ECM proteins. The upregulation of these proteins indicates that GS-Rb1 improves the signalling processes involved in tissue regeneration and enhances the mechanisms of tissue repair in tissues subjected to mechanical trauma. Therefore, TGF-β1/Smad3 signalling is essential for the GS-Rb1-mediated repair of mechanically injured tissue, emphasizing its potential as a therapeutic target for enhancing tissue regeneration and repair.

Furthermore, a proteomic study reported that small extracellular vesicles (sEVs) from fibroblasts contain TGF-β1 and other differentially expressed proteins involved in fibroblast regulatory signalling pathways. Thus, fibroblast sEVs may influence the pathogenesis of SUI via TGF-β1 and other proteins (Sun et al. 2021). Similarly, miR-328a-3p from EVs produced from bone marrow-derived BMSCs is transferred into fibroblasts to regulate ECM metabolism through the sirtuin 7/transforming growth factor β1 signalling pathway (Zhang et al. 2020a). In addition, earlier research has shown that TGF-β1, which is a key factor involved in the fibrosis of many tissues, also helps MDSCs become fibroblasts and accelerates the fibrosis process (Wang et al. 2011). In an animal model of SUI, reducing Smad3 expression can decrease the fibroblast development of MDSCs and enhance the ability of the urethral sphincter to recover (Wang et al. 2011). These findings may provide an innovative approach for treating SUI and improve the results of MDSC implantation therapy in female SUI patients.

Several SUI-related animal processes, such as age-related urethral sphincter muscle fibrosis in rabbits, are associated with dysregulated Wnt-catenin and TGF-β signalling (Rajasekaran et al. 2019). These two pathways trigger the fibrosis and atrophy of the urethral sphincter muscles with ageing and multiparity. Aged animals may exhibit impaired urethral closure function. Furthermore, previous studies linked mechanical injury to the inhibition of Nrf2/antioxidant response element (ARE) signalling, which mediates transforming growth factor (TGF)-1/Smad3 signalling. These findings suggest that trauma-induced SUI may involve this pathogenic mechanism (Tang et al. 2017a). However, direct proof of the mechanical injury mechanisms in nerve cells is unavailable. Hence, more research is needed to determine the underlying cause of pudendal nerve injury in SUI patients.

Previous research has shown that urethral muscle loss may compromise structural and functional integrity. Li and others (Li et al. 2012) reported that UI rats presented less fragmentation and disorganization of total and striated muscles. They also reported that muscle and epithelial cells increased the expression of TGF-β1 and P-Smad2. Therefore, the TGF-β1/Smad pathway may also involve the release of MMP—9 from muscle cells and the urethral lining. Initial studies revealed that relaxin reduced overall TGF-β1 levels, particularly in fibroblasts from women with SUI. Briefly, relaxin increased the level of active TGF-β1 in the supernatant. During the proliferative phase of the menstrual cycle, its level in the ECM of fibroblasts isolated from women with SUI was reduced. Changes in active TGF-β1 levels in relaxin-treated fibroblasts from women with SUI may be explained by the increased secretion of active TGF-β1 from fibroblasts and/or increased MMP-2 and NE activity, which might release active TGF-β1 from the ECM (Wen et al. 2008). Therefore, pregnancy-induced increases in relaxin levels may lead to markedly reduced ECM levels of both total and active TGF-β1. For women who are susceptible to SUI, this change might alter matrix synthesis and impair the tissues supporting the pelvic region.

Moreover, recent investigations have indicated that suppressing the TGF-β1/Smad3 signalling pathway results in a metabolic imbalance in the ECM, leading to the pathogenesis of SUI (Li et al. 2022a). Furthermore, a study revealed that the anterior vaginal wall of SUI rats presented considerably higher expression of TβR-2 and Smad7 than that of blank-treated and control rats. On the other hand, Smad3 expression was significantly decreased (Wang et al. 2015). Compared with those of control and blank-treated rats, the anterior vaginal wall collagen fibres of SUI rats were significantly impaired (Wang et al. 2015). These findings suggest that the pathological process of SUI may involve TGF-β/Smad3 signalling.

Because bladder tissues cannot easily be collected in clinical trials, animal investigations have been conducted to study the molecular mechanism of SUI. In a parturition-induced SUI rat model, an oligo microarray analysis revealed increased expression of genes involved in inflammation (Smad2/TGF-B), smooth muscle regulation (RGS2), and collagen metabolism (MMP13) (Lin et al. 2009). The authors concluded that inflammation, smooth muscle inhibition, and collagen breakdown all play significant roles in the development of SUI (Lin et al. 2009).

## The roles of other cytokines in the progression of POP

### Chemokines

Darzi and colleagues reported that other cytokines, known as chemokines, play crucial roles in the progression of POP (Darzi et al. 2023). Substantial evidence indicates that POP in Loxl1 knockout (KO) mice is caused primarily by injuries sustained during vaginal pup delivery (Liu et al. 2004). In addition, compared with wild-type (WT) mice, pregnant and postpartum Loxl1 KO mice presented altered expression and concentrations of inflammatory cytokines in the vagina, urethra, bladder, and rectum (e.g., chemokine (C–C motif) ligand-7 (CCL7) and chemokine (C-X–C motif) ligand 12 (CXCL12) in response to tissue injury. These findings suggest that these mice are more susceptible to childbirth injuries, especially to injuries to the urethra and vagina (Couri et al. 2017). This study led us to conclude that LOXL1 deficiency is particularly important in the repair process following an injury, such as that associated with childbirth. The altered expression and concentrations of inflammatory chemokines (e.g., CCL7 and CXCL12) in Loxl1 KO mice indicate that these mice have a compromised ability to respond to and repair tissue damage in the vagina, urethra, bladder, and rectum. This impaired repair mechanism increases the susceptibility of Loxl1 KO mice to injuries during childbirth and indicates the critical role of LOXL1 in maintaining tissue integrity and facilitating effective healing postinjury.

Furthermore, hyaluronan synthase 2 (HAS2) plays a crucial role in the synthesis of HA, thus profoundly affecting the overall structure and function of the ECM(Skandalis et al. 2020). Recent bioinformatics studies revealed that HAS2 is associated with immune cell activation, chemotaxis, and cytokine release in individuals with POP and is implicated in ECM functions. Chemokine signalling, leukocyte migration, cell chemotaxis, cytokine production regulation, and cytokine‒receptor interactions are among the pathways through which these processes occur (Wu et al. 2024a). Thus, regulating HAS2 expression and activity can influence tissue repair (ECM) and inflammatory responses (chemokine pathways) in POP progression, suggesting that HAS2 is a potential POP treatment strategy. Similarly, another bioinformatics investigation revealed that POP-associated DEGs (CCL2) are enriched in the immune response, indicating that they may play crucial roles in the formation and progression of POP (Zhou et al. 2018).

## The roles of other cytokines in the progression of UI

### Chemokines

The effect of parturition on chemokine homing factor expression in the VD model of SUI was documented by Lenis and colleagues in a prior study. In virgin and postpartum rats, VD upregulated urethral CCL7 expression immediately after injury (Lenis et al. 2013). Furthermore, the authors reported that hypoxia-inducible factor-1α (HIF-1α) and vascular endothelial growth factor (VEGF) were upregulated exclusively in virgin rats after VD. Compared with that in virgin rats without VD, CD191 (CCR1) expression was immediately upregulated in postpartum rats without VD. Conversely, CD195 (CCR5) was upregulated in virgin rats three days after VD compared with virgin rats without VD. Virgin rodents exhibited delayed upregulation of CD193 (CCR3) and CXCR4 seven days after VD. CXCL12 was upregulated in virgin rats three days after VD compared with rats with successive VD, indicating that VD alone stimulates the expression of several types of chemokines and chemokine receptors that are involved in stem cell homing and the response to tissue injury. Furthermore, the expression of CD191 is influenced by pregnancy and parturition, which is independent of VD. Therefore, the urethral expression of factors that are involved in the tissue response to inflammation and repair is influenced by pregnancy and parturition.

## Pro- and anti-inflammatory cytokines as therapeutic targets in POP

### Vaginal mesh

Surgical mesh material is used to treat SUI and POP in female patients (Mangir et al. 2020). Vashaghian and colleagues (Vashaghian et al. 2019) postulated that mechanical stimulation could enhance the regenerative capacity of fibroblast-seeded electrospun membranes. Briefly, electrospun scaffolds were created with a PCL/PLGA blend and seeded with fibroblasts from patients with POP. Once the cell-scaffold constructions reached confluence, they were exposed to cyclic strain for 24 and 72 h. Mechanical strain reduces the ability of cells to differentiate into myofibroblasts. Mechanical stimulation might help fibroblast-seeded electrospun membranes heal better by increasing the expression levels of genes involved in generating matrices, remodelling (α-SMA, TGF-β1, and MMP-2), and decreasing inflammation (IL-1β, TNF-α, and IL-8) (Table 1). Indeed, mechanical cues play important roles as signalling cues in the native ECM. In addition to mechanical cues, investigating other biophysical cues, such as scaffold stiffness, surface topography, and even electrical stimulation, for electrospun scaffolds is important (Vashaghian et al. 2018).

**Table 1:** The pro- and anti-inflammatory cytokines in POP clinical and preclinical research. Symbols ↑ indicated up-regulation, ↓ down-regulation, and ↔ no change

Mesh/inhibitor/influencer	Target	Study type	Site of insertion/Sample	Outcome	References
S-nitros- oglutathione (GSNO) coating PP mesh	IL-1 and TNF	Preclinical	The abdominal wall between the hypodermis and the anterior fascia of the abdominal musculature	IL-1 and TNF↓	(Prudente et al. 2017)
Small intestinal submucosa (SIS)/PP mesh	IL-1β and IL-6	Preclinical	Pelvic sub- mucosa	IL-1β and IL-6↓	(Ge et al. 2016)
standard-weight (SW) and lightweight (LW) PP meshes	IL-1 and TNF-α	Preclinical	Abdomen	IL-1/TNF-α↔	(Bronzatto and Riccetto 2018)
Restorelle mesh (Coloplast, Humblebaek, Denmark)	IL-10	Preclinical	Vagina	IL-10↑	(Brown et al. 2015)
Bacterial cellulose (BC) mesh	IL-2a/IL-4/IL-10 and TNF-α	Preclinical	Vagina	IL-4↓ and IL-2a/IL-10 and TNF-α↔	(Ai et al. 2020a)
Poly L-lactic acid-co-poly e-caprolactone nanofibrous mesh with endometrial mesenchymal stem/stromal cells	IL-1β/TNF-α/IL-4a/IL-6	Clinical	Endometrial biopsies	IL-1β and TNF-α↔, IL-4a/IL-6↑	(Mukherjee et al. 2020)
Chitosan biomaterial and PP mesh	IL-6/IL-10	Preclinical	Mucosa of the anterior vaginal wall	IL-6↑and IL-10↓	(Stangel-Wójcikiewicz et al. 2024)
Titanized polypropylene lightweight mesh (TiLOOP Mesh)	IL-4/IL-10/IL-12/TNF-α	Preclinical	Vagina	IL-10 and TNF-α↓ and IL-4 and IL-12a↔	(Ai et al. 2020b)
Core-sheath polystyrene/ gelatin electrospun nanofiber mesh	IL-10/IFN-γ	Preclinical	Vagina	IL-10 and IFN-γ↑	(Ge et al. 2015)
PP mesh	TGF-β	Preclinical	Vagina	TGF-β↑	(Artsen et al. 2019)
Laparoscopic pelvic floor reconstruction without mesh	IL-1p3/TNF-α	Clinical	Blood	IL-1p3 and TNF-α↓	(Wu et al. 2022)
Pessary insertion	IL-1β/IL-4/IL-10/IL-12p70/TNF-α/IFN-γ	Clinical	Vaginal swab samples	IL-1β/IL-4/IL-10/IL-12p70/TNF-α/IFN-γ↑	(Kim et al. 2023)
Local estrogen therapy (LET)	IL-1/IL-6/IL-8/IL-16 and IFN-γ	Clinical	Vaginal tissue samples	IL-6/IL-16 and TNF-γ↑ and IL-1 and IL-8 ↔	(Tyagi et al. 2019)
MiR-138	IL-1β	Preclinical	Bone marrow-derived mesenchymal stem cells	IL-1β↓	(Zhao et al. 2021a)
Pue-loaded GelMA (Pue@GelMA)	IL-3/IL-6/TGF-β1/TNF-α > 1	Preclinical and clinical	rabbit abdominal wall tissue samples and anterior and posterior vaginal walls of human tissue	IL-3, IL-6↓, TNF-α, TGF-β1 > 1↑	(Qin et al. 2020)
Age-related enzyme klotho	IL-6	Clinical	Pelvic floor fibroblasts	IL-6↓	(Qiu et al. 2019)
Knockdown of early growth response 2	TGF-β	Clinical	Anterior vaginal wall tissues	TGF-β↑	(Jin et al. 2024)
Poly (lactic-co-glycolic acid/ polycaprolactone (PLGA /PCL)	IL-1β/TNF-α/IL-8/ TGF-β	Clinical	Cell-seeded samples	IL-1β, TNF-α, IL-8↓ and TGF-β↑	(Vashaghian et al. 2019)
PP mesh	IFN-ϒ	Preclinical	Blood and Vaginal tissue	IFN-ϒ↑	(Liang et al. 2022)
Pelvic floor electrical stimulation (ES)	IFN-γ, IL-2/IL-4, IL-10, IL-17 A, and TNF-α	Clinical	Blood	IFN-γ, IL-2, IL-4, IL-10, IL-17 A, and TNF-α↓ and IL-6↑	(Zhang et al. 2022b)
Exosomes derived from BMSCs treated with TNF- α	IL-6/TNF-α	Clinical/preclinical	Vaginal wall tissues	IL-6 and TNF-α↓	(Zhou et al. 2024)

In an attempt to reduce the inflammatory reaction following implantation, nitric oxide (NO) was adsorbed to the surface of the mesh, utilizing polyvinyl alcohol as a scaffolding transporter (Prudente et al. 2017). These meshes had a lower inflammatory (IL-1 and TNF) response than did the uncoated meshes; nevertheless, exogenous NO is cytotoxic (Prudente et al. 2017) (Table 1). In addition, Ai and colleagues reported that at 1 and 12 weeks after implantation, the TiLOOP light mesh group presented lower mRNA expression levels of TNF-α and IL-10 than did the Gynemesh PS group. These findings indicate that the anti-inflammatory properties of the TiLOOP light mesh reduce inflammatory cytokine levels (Ai et al. 2020b). Recent research has examined the effects of an anti-inflammatory Chinese traditional medicine-loaded gelatine methacryloyl (GelMA) hydrogel on local immune microenvironment regulation in a rabbit abdominal wall hernia model (Qin et al. 2020). The porous hydrogel stimulated the TGF-β/MMP pathway to support fascia regeneration by inhibiting neutrophil and eosinophil recruitment into the wound region. Furthermore, xenogeneic degradable meshes induce the release of IL-10, a suppressive and anti-inflammatory cytokine. In contrast, nondegradable prolene induces inflammatory signals, including TNF-α and interferon-gamma (IFN-γ), in a mouse model (Bø and Hilde 2007). Based on these characteristics, degradable meshes are less immunogenic than their nondegradable equivalents. As a result, a reduced chance of infection may exist and a particular antibacterial immune response may develop. At this point, whether the degradable nature, biological origin, or a combination of the two factors is responsible for the decreased immunogenicity is unclear.

**Fig. 6:**
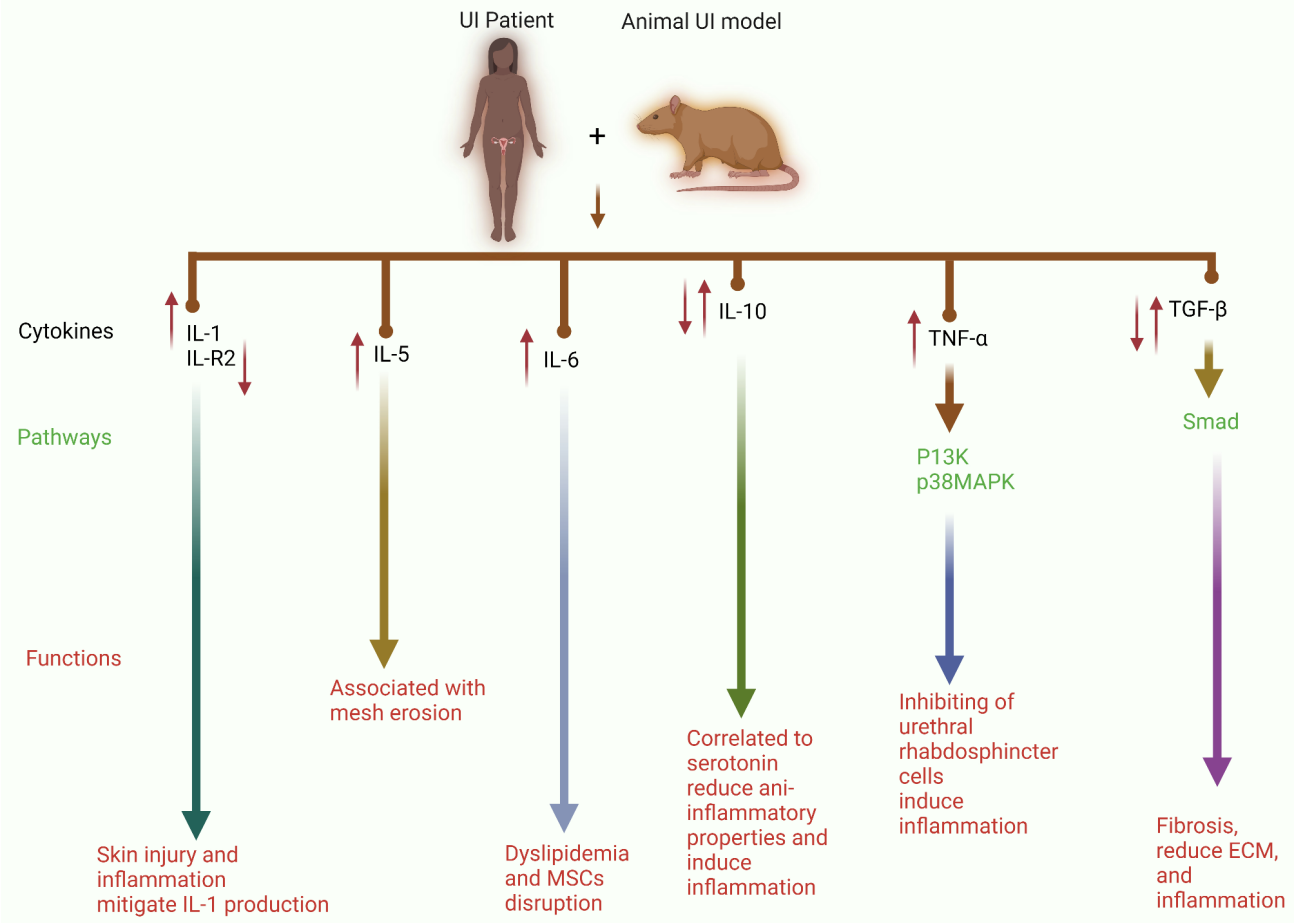
Pro- and anti-inflammatory cytokines significantly influence the development and progression of UI in patients and in a UI animal model. The release of large amounts of IL-1 induces skin injury and inflammation, whereas the downregulation of IL-R2 mitigates IL-1 production during UI. Mesh erosion is associated with the upregulation of IL-5, and this increase in IL-5 expression acts as a biomarker for UI. Moreover, IL-6 induces dyslipidaemia and disrupts MSCs. IL-10 expression is correlated with the levels of serotonin, which reduces the anti-inflammatory properties of IL-10 and increases the proinflammatory response. TNF-α inhibits urethral rhabdosphincter cells and induces inflammation via the P13K and p38MAPK pathways. Through the Smad pathway, TGF-β dysregulation has a dual role. These factors decrease the ECM and inflammation, whereas they accelerate the fibrosis process

Furthermore, the novel biomaterial Pue@GelMA reduces inflammation by preventing neutrophil and eosinophil aggregation and lowering the levels of IL-3 and IL-6 (Table 1). Moreover, a study proposed a treatment strategy for POP based on drug-loaded hydrogels that combine anti-inflammatory properties with tissue regeneration. A photo-crosslinked gelatine methacryloyl (GelMA) loaded with puerarin (Pue) inhibits neutrophil and eosinophil aggregation while promoting matrix remodelling through the TGF-β/MMP pathway in a rabbit POP model (Qin et al. 2020). Pue@GelMA decreased the levels of inflammatory cytokines (IL-3, IL-6, TNF-α, and TGF-β1) and ECM factors (COL-1, COL-3, MMP2, and MMP9) while increasing the levels of collagen types 1 and 3. An immunohistochemical study demonstrated that Pue@GelMA successfully altered the immunological milieu by reducing neutrophil and eosinophil aggregation and altering the ECM collagen distribution (Qin et al. 2020). This hydrogel method enhanced fascia regeneration and markedly decreased implantation-related inflammatory responses in vivo. Pue@GelMA therapy promoted the host’s anti-inflammatory milieu by increasing TGF-β1/TNF-α gene expression. Pue@GelMA thus promotes the equilibrium of TGF-β1 and TNF-α, ECM production, fascia tissue regeneration, and subsequent healing (Fig. 6) (Qin et al. 2020). Therefore, early immune modulation contributes to ECM remodelling and spatial and temporal muscle fascia restoration, indicating the potential for hernia repair. Moreover, in a POP mouse model, Mukherjee et al. synthesized an electrospun NF poly L-lactic acid-co-poly-caprolactone (PLCL) mesh loaded with endometrial mesenchymal stromal cells (MSCs) to modify foreign body reactions using multiple strategies. At six weeks postimplantation, electrospun NF meshes loaded with MSCs were used to treat POP in mice and significantly increased angiogenesis, cell adhesion, and the activity of the immune system. Additionally, the expression of pro- and anti-inflammatory genes (IL-4a and IL-6) increased, aiding in reducing inflammation and accelerating the healing process (Mukherjee et al. 2020). All the above studies suggest an excellent chance of a successful implant and a viable alternative to surgical operations. Therefore, until such data become accessible, only clinical studies approved by the local ethics committee should employ these latest transvaginal meshes.

Current research has investigated strengthening the pelvic floor through the use of bioactive tissue-engineered meshes. In their study, Darzi et al. examined the advantages of tissue engineering techniques that employ uterine endometrium-derived mesenchymal stem/stromal cells (eMSCs) in combination with gelatine (G) and degradable poly L-lactic acid-co-poly ε-caprolactone (PLACL) to create a composite electrospun nanofibrous mesh (P + G nanomesh) and assessed the immunomodulatory mechanism at the material interfaces (Darzi et al. 2023). This study emphasized critical acute and chronic inflammatory markers, as well as remodelling factors, determining the outcome of mesh surgery. The authors postulated that the foreign body response (FBR) at the host interface, which is associated with mesh complications, is mitigated by the bioengineered construct and that mesh integration is improved. At six weeks postimplantation, the authors documented that eMSC-based nanomesh substantially increased the expression of genes associated with ECM synthesis and cell adhesion, such as Tgfb1 and Tgfbr1, and other genes related to angiogenesis, such as CXCL12 (Darzi et al. 2023). These findings indicate that cell-based tissue-engineered structures may reduce the FBR response generated by biomaterial implants. From a medical perspective, this design addresses current deficiencies in surgical outcomes by controlling the immune response and promoting angiogenesis and ECM formation during the acute and chronic phases of the FBR.

More recently, in animal models of POP, a study investigated tissue regenerative design characteristics such as degradability, porosity, and angulation to create alternative degradable melt electrowritten (MEW) structures for surgical purposes (Paul et al. 2024). The anti-inflammatory phenotype is characterized by a higher ratio of anti-inflammatory CD206 + M2 macrophages/proinflammatory CCR7 + M1 macrophages in the FBR, which is influenced by the pattern and geometry of the multiple-layer MEW implants. Briefly, the study compared the immune responses of 22.5°2P and 90°2P meshes, with a particular emphasis on gene expression associated with the immune response, cell adhesion, and ECM. Proinflammatory cytokines (IL-1β, TNF-α, IL-13, and IL-6), cell adhesion markers (Vcam1, Icam1, and Cd44), and chemokines (CXCR3 and CXCL12) were upregulated in the smallest 22.5°2P meshes at 1 week, but they were downregulated by 6 weeks. In contrast, the 90°2P meshes exhibited sustained high expression of proinflammatory cytokines (IL-6 and IL-1) and chemokines (CXCR3 and CXCL12), as well as upregulation of the anti-inflammatory molecule IL-10. Initially, 22.5°2P meshes exhibited acute inflammation and upregulated expression of monocyte (CD68) and myeloid lineage genes (Cd11b). However, these effects were subsequently suppressed. Proinflammatory factors (IL-6, IL-1, and CXCR3) were persistently upregulated in 90°2P meshes at 6 weeks, suggesting that the FBR was ongoing. The smallest mashes facilitated tissue integration by facilitating the transition from M1 to M2 macrophages. This transition was accompanied by substantial early expression of Col3a1 and Fgfr3, followed by Col1a1 at 6 weeks (Paul et al. 2024). These results suggest that 22.5°2P meshes modulate the immune response and enhance tissue regeneration more effectively than 90°2P meshes do. The validation of these data using two preclinical models indicates the role of mesh geometry in modulating the implant/tissue reaction, which is a pivotal step for next-generation pelvic floor implants.

Exposure to mesh induces inflammatory reactions because asymptomatic mesh infection prevents the mesh from integrating with its surroundings (Mangir et al. 2020). Fibrosis, which is induced by elevated TGF-β1 levels, appears to be a contributing factor in explants from women experiencing pain and exposure to mesh issues (Artsen et al. 2019). Furthermore, the PP mesh inserted into the vagina promotes constant inflammation at the mesh‒tissue interface. In a diabetic mouse model of POP, mesh-associated inflammation was enhanced by the upregulation of cytokines, including IFN-γ, which led to a dysregulated macrophage response (Liang et al. 2022). Therefore, after mesh implantation, women with uncontrolled diabetes are more likely to experience mesh challenges. Although PP meshes with stable pores and ultralight weights may be able to evoke a more positive foreign body response because of the prolonged inflammation associated with mesh utilization, new biomaterials with lower immunogenicity are recommended. In addition, a better understanding of how the cytokine response to the mesh affects macrophages will help in the creation of cell-based therapies that will improve the outcomes of vaginal implants for diabetic women. Table 1 provides a comprehensive overview of the pro- and anti-inflammatory cytokines used as therapeutic targets in preclinical and clinical research on POP.

### Other inhibitors

Recent research has investigated the impacts of local oestrogen on collagen/elastin biogenesis and immunological markers (e.g., IL-6, IL-16, and TNF-γ) (Table 1) in women with POP (Tyagi et al. 2019). This study provides evidence that local oestrogen therapy can effectively inhibit ECM degradation in hypoestrogenic women by activating the immune system in the local vaginal environment. However, future studies still need to evaluate the clinical significance of these findings. In addition, previous research revealed that treating POP patient fibroblasts produced in vitro with klotho (an age-related enzyme) greatly increased cell proliferation, reduced ROS levels, and decreased IL-6 release (Qiu et al. 2019) (Table 1). These results suggest that klotho may increase the resistance of pelvic floor fibroblasts in POP patients to oxidative stress and induce an anti-inflammatory response.

## Pro- and anti-inflammatory cytokines as therapeutic targets in UI

### Innovative methods for treating UI and physical activity (yoga)

Recent research using electrophysiological stimulation of the lower pelvic floor muscles has examined the biological pathways implicated in SUI therapy. Sports widely recognize electrophysiological stimulation for its ability to promote tissue regeneration in athletes by reducing pain, increasing local blood circulation, and relaxing muscles after activity. These data have been applied to preclinical investigations of SUI treatment in animal models. In mice, VD triggered symptoms similar to those of SUI. Afterwards, electrophysiological stimulation was applied for a maximum of seven days of follow-up (Min et al. 2017b). The effects of VD and its regeneration via electrophysiological stimulation in this SUI model were studied, and standard clinical measurements were used. Thus, the maximum bladder capacity and LPP were calculated. Voiding during sneezing was measured as an alternative to a pad test (Min et al. 2017b).

Electrical stimulation markedly enhanced the maximal bladder capacity and LPP, and reduced urine loss during sneezing in incontinent mice. This animal model also enabled histological examinations of the treated tissue. Here, strong-to-notable induction of type I collagen expression was observed, depending on the electrical stimulation regimen. VD was associated with a significant reduction in TGF-β1 expression, as well as the activation of SMAD-2 and SMAD-3. Nevertheless, following electrophysiological stimulation, SMAD phosphorylation was dramatically increased (Min et al. 2017b). Since activated phospho-SMAD-2 (p-SMAD2) and p-SMAD-3 are particular intracellular proteins that are involved in the signal transduction of the TGF-ß pathway, a decrease in their levels is not surprising (Table 2). TGF-β1 also increases the production of ECM components and is a key regulator that affects how cells grow and differentiate (Zhang et al. 2014). This mechanism also suggests that TGF influences SMAD phosphorylation in this SUI model.

**Table 2:** The pro- and anti-inflammatory cytokines in UI clinical and preclinical research. Symbols ↑ indicated up-regulation, ↓ down-regulation, and ↔ no change

Inhibitor/influencer	Target	Study type	Sample	Outcome	References
Lactobacillus rhamnosus GR-1 and Lactobacillus reuteri RC-14	TNF-α/IL-6, IL-8/IL-10 /IL-12 (p70)	Clinical	Urine and blood	TNF-α, IL-6, IL-8, IL-10 and IL-12 (p70) ↓	(Anukam et al. 2009)
Yoga	IL-6/TNF-α	Clinical	Blood	TNF-α↓ and IL-6↔	(Tenfelde et al. 2020)
Pessary-wearing	IL-6/TNF-α/IL1β	Clinical	Vaginal swabs	IL-1β/IL-6 and TNF-α↑	(Ramaseshan et al. 2022)
Physical rehabilitation therapy combined with shixiao powder and siwu decoction	IL-10	Clinical	Medical records	IL-10↓	(Han et al. 2018)
Punicalagin (PUN; 2,3-hexahydroxydiphenoyl-gallagyl-D- glucose)	TGF-β	Preclinical	Vaginal tissue	TGF-β↑	(Tang et al. 2017b)
Electrical stimulation	TGF-β	Preclinical	Vaginal tissue	TGF-β↑	(Li et al. 2019b)
Electrical stimulation	TGF-β	Preclinical	Anterior vaginal wall specimens	TGF-β↑	(Min et al. 2017b)
Dimethyl fumarate (DMF)	TGF-β	Preclinical	Vaginal wall tissue and blood	TGF-β/Smad3↓	(Liu et al. 2022)
Electrical stimulation	TGF-β	Clinical	Female vaginal wall fibroblasts cells	TGF-β↑	(Li et al. 2019a)
Puerarin pretreatment	TGF-β	Preclinical	Anterior vaginal tissues	Nrf2/TGF-β1↑	(Li et al. 2022b)
Periurethral and intravenous injection of adipose-derived stem cells	TGF-β1	Preclinical	Urethra specimen	TGF-β1↓↑	(Li et al. 2022a)
MSCs	CCL7 and CCR1	Pre-clinical	Periurethral tissues	CCR1-overexpressing MSCs and CCL7 can increase engraftment of MSCs and promote the functional recovery of simulated birth trauma-induced SUI in rats	(Jiang et al. 2020)
CXCL12 injection	CXCL12	Pre-clinical	Periurethral and urogenital tissues	CXCL-12 was feasible and improved the UI	(Zambon et al. 2018; Khalifa et al. 2020)
ADSCs injection	CXCL12/CXCR4	Pre-clinical	Urethral tissues	The CXCL12/CXCR4 axis is involved in the migration of ADSCs and may play a role in the migration of ADSCs in SUI	(Li et al. 2024a)
Chitin-based hydrogel loaded with fibroblast growth factor (bFGF)/SDF-1(CXCL12)	CXCL12	Pre-clinical	Urethral tissue	It slowly releases factors and promotes the homing of MSCs in vivo, which can improve the local microenvironment, increase collagen deposition, repair the tissue around the urethra and finally improve SUI	(Yang et al. 2023)

In addition, a recent study showed that the mRNA levels of TGF-β1 and type I and type III collagens were increased by electrophysiological stimulation of human vaginal wall-derived fibroblasts (Li et al. 2019a). Interestingly, the expression of integrin β1 (CD29), TGF-β1, and type I collagen is considerably lower in the fibroblasts of SUI patients than in those of donors without SUI (Li et al. 2019a). These findings reveal that fibroblasts from surrounding sites also present changes in collagen and TGF production and regulation in the urethra. Pretreating these fibroblasts with an antibody against integrin β1 decreases their response to electrophysiological stimulation. These findings suggest that the integrity of cell‒matrix connections may influence susceptibility to electrophysiological stimuli. In this case, intracellular integrin signalling could be important.

Similarly, Li and colleagues reported that electrical stimulation (ES) increased the amount of collagen, activated integrin β1, regulated TGF-β1 to prevent apoptosis, and increased the calcium level in podocytes (Li et al. 2019b). A recent longitudinal study at Peking University International Hospital in Beijing explored the effects of pelvic floor ES on the vaginal microbiota and inflammatory responses in patients with POP and UI (Zhang et al. 2022b). The findings revealed a remarkable increase in the abundance of *Lactobacillus* spp. following ES treatment, accompanied by a reduction in the microbial diversity. Notably, the long-term cohort exhibited the significant downregulation of critical proinflammatory cytokines, including IFN-γ, IL-2, IL-4, IL-10, IL-17 A, and TNF-α. However, the short-term cohort presented elevated IL-6 levels at 7 h after treatment (Zhang et al. 2022b). IL-6 is instantaneously produced in response to infection and tissue damage, and contributes to the host’s defence against emergent stress by activating the acute phase and immune response (Tanaka et al. 2018). The study revealed that IL-6 levels increased significantly after a single ES intervention, unlike the nonsignificant changes observed with repeated ES interventions, while the levels of other cytokines paradoxically decreased. These findings suggest that pelvic floor ES may not induce local inflammation and could suppress inflammatory cytokines. This process warrants further investigation to explore additional clinical applications of ES. Additionally, the results indicate that transvaginal ES may facilitate the restoration and maintenance of a *Lactobacillus*-dominated vaginal microbiota, potentially reducing the risk of vaginal inflammation and enhancing pelvic health. Consequently, ES has therapeutic potential as a clinical treatment for SUI. However, further research is needed to elucidate the molecular mechanisms underlying the enhanced wound healing observed with electrophysiological stimulation.

In a study by Tenfelde et al. that was conducted with women who lived with UUI, yoga increased the quality of life of women and reduced the incontinence symptom burden (Tenfelde et al. 2020) (Table 2). Furthermore, it significantly reduced objective measures of inflammation (TNFα levels). This research shows that yoga improves quality of life postpartum, which is a difficult period for new mothers.

### Pharmacological therapy

Puerarin (7,4-dihydroxy-8-D-glucosylisoflavone, C21H20O9), a C-glycoside of daidzein, is one of the essential bioactive components extracted from kudzu root. The strong antioxidant activity of puerarin is among its many pharmacological properties (Zhou et al. 2019). A recent study revealed that puerarin protected fibroblasts from mechanical injury-driven ECM remodelling through the nuclear transcription factor 2 (Nrf2)/TGF-β11 pathway. These findings suggest that suppressing Nrf2 signalling may be critical for the ECM remodelling produced by mechanical damage (Li et al. 2022b) (Table 2). Tang and colleagues reported the potential therapeutic impact of punicalagin, also known as PUN (P2,3-hexahydroxydibenzoyl-gallagyl-D-glucose), on SUI following mechanical damage in mice. This process might also be connected to Nrf2 (Tang et al. 2017b). Furthermore, dimethyl fumarate (DMF) also positively affects SUI after mechanical damage in mice. In a VD-induced SUI mouse model, researchers reported that DMF could improve urethral closure dysfunction by blocking ECM remodelling. The Nrf2-TGF-1/Smad3 pathway and its downstream tissue growth factors mediate this treatment-induced association with DMF (Liu et al. 2022). Therefore, the research mentioned above provides novel ideas for treating SUI caused by mechanical trauma, such as childbirth, and suggests that targeting the inflammatory framework may be a useful therapeutic strategy.

### IL-5 antagonist treatment

Two patients with symptomatic eosinophilic cystitis (EoC) were effectively treated with benralizumab (an interleukin 5 antagonist). The first patient, a 78-year-old woman, presented with severe incontinence, pelvic pain, and other symptoms that did not improve with different medication regimens. The patient’s symptoms and quality of life significantly improved after starting benralizumab. However, her EOC was not completely cured. A sample obtained nine months into therapy revealed rare eosinophils but persistent cystitis (Cooke and Cooke 2020). Therefore, additional confirmation through clinical trials is still needed.

### Cellular therapy and cytokines (chemokines)

Stem cell treatment (SCT) appears to be a promising approach for restoring urethral sphincter function in patients with SUI (Aragón et al. 2018; Barakat et al. 2020; Klapper-Goldstein et al. 2022; Mariotti et al. 2023), and Klapper-Goldstein et al. (Klapper-Goldstein et al. 2022) reported that this therapy is safe, effective, and promising for the future in a recent systematic evaluation of the clinical studies available on SCT for SUI in women. Mariotti and colleagues (Mariotti et al. 2023) conducted a systematic review and meta-analysis of 12 clinical studies, revealing a 41% mean rate of continence recovery with the use of autologous muscle-derived (MDSC) or adipose-derived stem cells (ADSCs). However, cell treatment still encounters some challenges (Schmid et al. 2021), and an opportunity for progress seems to exist.

These MSCs are likely crucial in the engraftment of MSCs (Honczarenko et al. 2006). In addition, the chemokine/chemokine receptor axis, C-C motif ligand 7 (CCL7)/C-C motif receptor 1 (CCR1), is involved in the recruitment of MSCs in urethral and vaginal tissues following postpartum injury (Huang et al. 2010; Jiang et al. 2020). Modified bone marrow-derived MSCs designed to overexpress CCR1 were injected into SUI rats through the tail vein, and 2 µg of the active CCL7 peptide was injected in the tissue surrounding the urethra. Compared with the control rats, the rats in the SUI group presented the highest percentage of successfully transplanted MSCs in the injured tissue and the most significant level of functional recovery from SUI. However, the authors examined the transplantation rate and urethral recovery of MSCs only one week after the rats had been injured during childbirth, and the long-term outcomes were not considered. Thus, future research should focus on evaluating the long-term efficacy and stability of MSC transplantation in SUI recovery.

CCL7 can also recruit MSCs to an injured area in the myocardium, where they engraft, produce growth factors, facilitate healing from injury, and improve functional recovery (Schenk et al. 2007). In a mouse model of simulated birth trauma-induced UI (Hijaz et al. 2013), Hijaz and coworkers investigated the expression of CCL7. They reported the overexpression of urethral CCL7 after VD but not pudendal nerve transection, suggesting that nerve injury does not contribute to CCL7 overexpression.

In addition, cells create a chemokine called CXCL-12/SDF-1 as a substitute that assists in tissue regeneration in injured areas (Sun et al. 2019). CXCL-12 was previously employed as a local treatment to promote the regeneration of urogenital tissues. In a study involving nonhuman primates (NHPs), open radical prostatectomy (ORP) resulted in persistent erectile and urinary tract dysfunction. The feasibility of periurethral injection of CXCL-12 has been documented, resulting in enhancements in both erectile dysfunction and UI (Zambon et al. 2018). This finding implies that the NHP model can be effectively employed to evaluate novel therapeutic approaches for these conditions.

Furthermore, the continence function of the SUI female rat model improved upon the injection of the CXCL12 overexpression plasmid. A higher vascular density and greater urethral sphincter muscle mass suggest that this plasmid represents a possible therapeutic option for SUI (Khalifa et al. 2020). In a recent study, Li and others reported that the urinary function of SUI model rodents was enhanced by the intravenous injection of ADSCs, with CXCR4-positive ADSCs being detected in urethral tissues (Li et al. 2024a). This study examined the CXCL12/CXCR4 axis in ADSC homing and revealed that CXCR4 and CXCR7 expression increased following CXCL12 stimulation and hypoxic conditioning. The mRNA levels of CXCR4 and CXCR7 were significantly reduced upon their knockdown (Li et al. 2024a). The CXCL12/CXCR4 axis was essential for ADSC migration, as evidenced by the increased phosphorylation of JAK, AKT, and ERK in ADSCs stimulated with CXCL12. The inhibition of migration was achieved by silencing CXCR4 or blocking JAK and AKT signalling but not by silencing CXCR7 or blocking ERK. Therefore, the CXCL12/CXCR4 axis is crucial for ADSC migration and may contribute to SUI. In the above studies, we mentioned that inducing a stem cell homing effect is a good choice for the treatment of SUI. In addition, a novel injectable hydrogel composed of beta-chitin (bFGF (fibroblast growth factor)/SDF-1/chitin hydrogel) has been created by researchers (Yang et al. 2023). Briefly, hydrogels with a 3% concentration have rapid gel-forming characteristics, which can immediately stabilize CXCL12 inside the hydrogel and delay its release in vivo. In the early stages, the stiffness of the hydrogel provides urethral support, similar to tension-free vaginal tape obturator slings. Later, released factors induce BMSCs to home and differentiate into fibroblasts (Yang et al. 2023). The three-dimensional structure of hydrogels can stabilize BMSCs and create a cellular environment that stimulates fibroblast replenishment and collagen formation, repairing the supportive structure under the urethra and reducing SUI symptoms. Thus, a regenerative medicine approach based on a bFGF/SDF-1/chitin hydrogel could be an effective nonsurgical strategy to address clinical SUI.

In the above studies, stem cells exert their therapeutic effects primarily through the secretion of bioactive factors rather than through the cells themselves, underscoring the significant potential of homing factors such as CXCL12/CXC4, CCL7/CCR1, and other cytokines in regenerative medicine. Current treatments for UI are limited in efficacy, as they need to address the underlying pathophysiology or achieve comprehensive tissue repair. While substantial challenges exist and controlled clinical trials in UI patients are lacking, preclinical animal trials have produced promising outcomes. This emerging research suggests that stem cell therapy, mainly via the modulation of specific cytokines (homing factors), could revolutionize the treatment of voiding dysfunction and UI. Further investigations into these molecular mechanisms may lead to revolutionary advances in UI therapeutic techniques, serving as the basis for more effective and restorative interventions.

In the context of TNF responses to stem cell therapy in models of POP/UI, Zhou and colleagues reported that exosomes from TNF-α-stimulated BMSCs dramatically increase PFD in rats (Zhou et al. 2024). Compared with those of the controls, the vaginal wall of the POP model presented greater amounts of IL-6, TNF-α, and MMP2. Exosomes (Exos and TNF-Exos) were administered to PFD rats, resulting in increased void volume and bladder void pressure while decreasing peak bladder pressure and LPP (Zhou et al. 2024). TNF-Exos successfully restored elastin, collagen I, collagen III, IL-6, TNF-α, and MMP2 levels in the anterior vaginal wall. These findings suggest that the use of exosomes produced from TNF-α-treated BMSCs is promising as a novel therapeutic technique for alleviating PFD.

Furthermore, using an SUI rat model, researchers examined the effects of intravenous and periurethral ADSC injections on tissue healing and voiding function. This study revealed that in SUI rats, the two ADSC injection techniques caused varying degrees of healing of the urethral sphincter, secretion of cytokines (TGF-β1)/Smad pathway components, and rates of cell retention in the urethral tissues. Nevertheless, no significant differences were observed between the two techniques (Li et al. 2022a). The results of this study suggest that the TGF-β1/Smad pathway may be crucial for ADSC treatment of SUI. Notably, all existing studies on the use of ADSCs for treating SUI have been conducted at the preclinical level. To our knowledge, no clinical trials have investigated the modulation of cytokines in this context. Thus, the evidence to support the long-term efficacy and potential adverse reactions of this approach in clinical settings is insufficient. Future studies should focus on clinical trials to address these gaps.

## Conclusions and future perspectives

The complex interaction between proinflammatory and anti-inflammatory cytokines in patients with POP and UI provides a challenging vaginal microenvironment that can have positive and negative consequences. Proinflammatory cytokines, such as IL-1, IL-5, TNF, and IFN, are associated with the progression and development of POP and UI by promoting chronic inflammation and pain in the vaginal tissue of female patients. Moreover, some cytokines, such as IL-6, IL-10, and TGF, which are involved in tissue inflammation, remodelling, and repair, have dual effects on POP and UI. These cytokines can promote tissue healing and regeneration but can also exacerbate inflammation and fibrosis, thereby contributing to the progression of POP and UI in preclinical studies. Further research is needed to gain a precise understanding of how these “double-edged sword” cytokines affect illnesses such as POP and UI in a larger sample size of female patients.

To the best of our knowledge, women with POP and UI have not been the subject of extensive research on proinflammatory cytokines, such as members of the IL-1 family (IL-18 and IL-36), IL-17, and others, which are the primary causes of inflammation, or other classes of cytokines, such as CXC chemokine (CXCL1‒CXCL10) family members and their receptors, among others. Therefore, further research is necessary to gain a comprehensive understanding of CXC chemokines and proinflammatory cytokines in women with POP and UI.

Proinflammatory cytokine inhibitors, such as PP meshes and pharmacological interventions (drug therapy) and electrophysiological stimulation, that target specific cytokines are critical for inhibiting proinflammatory cytokine secretion while increasing anti-inflammatory cytokine secretion. Several investigations have shown that POP patients benefit from PP mesh implants because of the inhibition of proinflammatory cytokines. Furthermore, the majority of the related research has been preclinical studies on the favourable effects of meshes on reducing the levels of proinflammatory cytokines; hence, these findings provide us with a new perspective on the use of PP meshes in clinical trials. Nevertheless, future clinical trials employing these meshes are necessary to inhibit these proinflammatory cytokines in POP patients.

Additionally, electrophysiological stimulation is beneficial for UI patients. However, further research is necessary to fully comprehend the molecular mechanisms that underlie the enhanced wound healing resulting from electrophysiological stimulation. Furthermore, homing factors such as CXCL12/CXCR4 and CCL7/CCR1 may be helpful in regenerative medicine since stem cells secrete bioactive molecules. UI therapies often fail to correct the pathogenesis or regenerate tissue. Despite these limitations and a lack of controlled clinical trials in UI patients, preclinical animal research shows promise. Research suggests that stem cell therapy, particularly by regulating cytokines and homing factors, could revolutionize voiding dysfunction and UI treatment. Therefore, further investigation of these molecular mechanisms could improve UI treatment. These research findings will have profound consequences beyond academics. They can assist with treating female reproductive disorders such as POP and UI.

## Data Availability

No datasets were generated or analysed during the current study.

## Abbreviations

IL-1: Interleukin-1
IL-2: Interleukin-2
IL-4: Interleukin-4
IL-6: Interleukin-6
IL-7: Interleukin-7
IL-8: Interleukin-8
IL-10: Interleukin-10
IL-12: Interleukin-12
IL-15: Interleukin-15
IL-17: Interleukin-17
IL-18: Interleukin-18
IL-20: Interleukin-20
IL-21: Interleukin-21
IL-22: Interleukin-22
IL-23: Interleukin-23
IL-27: Interleukin-27
IL-33: Interleukin-33
IL-37: Interleukin-37
GM-CSF: Granulocyte–macrophage colony-stimulating factor
MIG: Monokine induced by gamma
IP-10: Interferon gamma-induced protein 10
TGF-β1: Transforming growth factor-beta 1
TNF-α: Tumour necrosis factor-alpha
IFN-γ: Interferon-gamma
JAK/STAT: Janus kinase/signal transducer and activator of transcription
SMAD2/3: Suppressor of mothers against decapentaplegic 2/3
NLRP3: Nucleotide-binding domain, leucine-rich-containing family, pyrin domain-containing-3
TIMP2: Tissue inhibitor of metalloproteinases 2
PCNA: Proliferating cell nuclear antigen
SOCS3: Suppressor of cytokine signalling 3
FVWFs: Female vaginal wall fibroblasts
BMSCs: Bone marrow mesenchymal stem cells
MDSC: Myeloid-derived suppressor cells
PCL/PLGA-BCP: Polycaprolactone/poly(lactide-co-glycolide) biphasic tricalcium phosphate
